# Identification of the Intrinsic Dielectric Properties of Metal Single Atoms for Electromagnetic Wave Absorption

**DOI:** 10.1007/s40820-021-00773-6

**Published:** 2021-12-11

**Authors:** Xinci Zhang, Yanan Shi, Jia Xu, Qiuyun Ouyang, Xiao Zhang, Chunling Zhu, Xiaoli Zhang, Yujin Chen

**Affiliations:** 1 Key Laboratory of In-Fiber Integrated Optics, College of Physics and Optoelectronic Engineering, Harbin Engineering University, Harbin, 150001 People’s Republic of China; 2 College of Materials Science and Chemical Engineering, Harbin Engineering University, Harbin, 150001 People’s Republic of China; 3 School of Materials Science and Engineering, Zhengzhou University, Zhengzhou, 450001 People’s Republic of China

**Keywords:** Metal single atoms, Dielectric loss behavior, NaCl-templating method, Lightweight absorbers, Honeycomb-like N-doped nanocarbons

## Abstract

Atomically dispersed metals on N-doped carbon supports (M–N_*x*_Cs) have great potential applications in various fields. However, a precise understanding of the definitive relationship between the configuration of metal single atoms and the dielectric loss properties of M–N_*x*_Cs at the atomic-level is still lacking. Herein, we report a general approach to synthesize a series of three-dimensional (3D) honeycomb-like M–N_x_C (M = Mn, Fe, Co, Cu, or Ni) containing metal single atoms. Experimental results indicate that 3D M–N_*x*_Cs exhibit a greatly enhanced dielectric loss compared with that of the NC matrix. Theoretical calculations demonstrate that the density of states of the *d* orbitals near the Fermi level is significantly increased and additional electrical dipoles are induced due to the destruction of the symmetry of the local microstructure, which enhances conductive loss and dipolar polarization loss of 3D M–N_*x*_Cs, respectively. Consequently, these 3D M–N_*x*_Cs exhibit excellent electromagnetic wave absorption properties, outperforming the most commonly reported absorbers. This study systematically explains the mechanism of dielectric loss at the atomic level for the first time and is of significance to the rational design of high-efficiency electromagnetic wave absorbing materials containing metal single atoms.

**Supplementary Information:**

The online version contains supplementary material available at 10.1007/s40820-021-00773-6.

## Highlights

- A general method was developed to fabricate a series of honeycomb-like N-doped nanocarbons (3D M–N_x_C) doped with metal single atoms (Mn, Fe, Co, Cu, or Ni) with a high yield.
- The intrinsic dielectric properties of 3D M–N_x_C were identified for the first time at the atomic-level, revealing that the introduction of metal single atoms greatly increases both conductive loss and polarization loss.
- 3D Mn–NC exhibited high-performance electromagnetic wave absorption at a low filler loading of 10 wt% outperforming most reported absorbers.

**Supplementary Information:**

The online version contains supplementary material available at 10.1007/s40820-021-00773-6.

## Graphical abstract

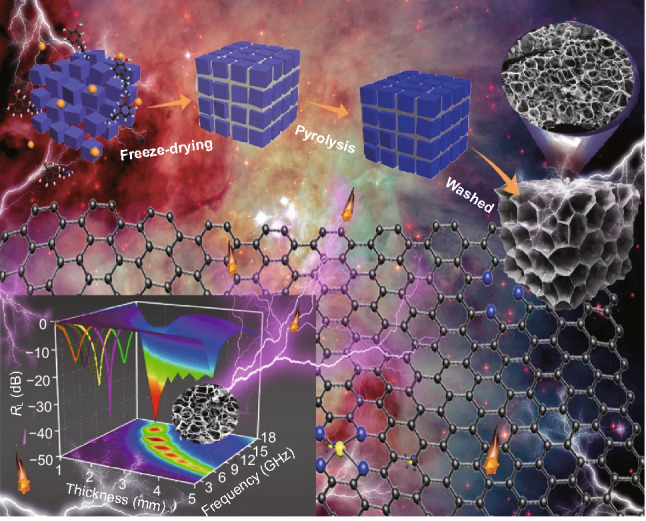

## Introduction

Atomic metal–N_x_ (M–N_x_) moieties anchored onto carbon support (M–N_x_C) can effectively modulate the electronic structures of adjacent carbon atoms, resulting in extraordinary physicochemical properties of M–N_*x*_Cs [1–4]. Moreover, well-defined single atomic sites provide an ideal platform for understanding the physicochemical mechanism at the atomic level [5–9]. In addition, carbon supports with high electrical conductivity are readily available for commercial use, which guarantees their applicability to various fields. For example, single silicon atoms anchored onto graphene were demonstrated to effectively induce surface plasmon resonances in graphene [6]. Single niobium atoms introduced into defective graphitic layers were found to break the asymmetry in the localized microstructure, thus causing an additional strong dielectric resonance behavior [7]. Single platinum atoms anchored on the surfaces of graphene nanosheets display a higher catalytic activity for methanol oxidation [9]. These pioneering studies suggest that unexpected physicochemical properties can be achieved using M–N_*x*_Cs.

Although important progress in M–N_*x*_Cs has been achieved, the mechanisms of several physical properties, such as the dielectric loss behavior related to electromagnetic wave absorption (EMW), are still unclear. In fact, systems consisting of metal single atoms anchored onto nanocarbons have potential application as lightweight materials for EMW absorption because M–N_*x*_Cs inherit the lightweight properties of nanocarbons. Furthermore, metal single atoms can be regarded as polarization centers, which can induce polarization loss for EMW absorption. Additionally, highly active single atoms can tune the electronic structure of adjacent carbons and can thereby regulate the conductive loss of M–N_*x*_Cs. However, a precise understanding of this structure–property relationship of M–N_*x*_Cs at the atomic level has not yet been attained. Moreover, different types of metal single atoms with similar moieties may have different effects on their dielectric behavior and EMW absorption properties. Thus, the influence of different types of metal single atom on the resulting properties needs to be uncovered. In addition, most strategies for the synthesis of M–N_x_C materials suffer from low yields and high costs, which hinder their practical applications [10–12]. Therefore, it is highly desirable to develop a general method to synthesize M–N_*x*_Cs in view of large-scale production.

In addition, the morphology of the absorbers has an important effect on their EMW absorption properties. In particular, 3D interconnected structures with open pores have shown good EMW absorption properties owing to their large surface area and abundant edge sites for improving the polarization loss as well as numerous pores for optimizing impedance matching properties [13–15]. Up to now, various 3D interconnected structures with open pores have been prepared for EMW absorption [16–18]. However, these 3D interconnected structures with open pores frequently contain functional zero- and/or one-dimensional nanostructures with a relatively large size, leading to a higher filler ratio in the matrix. If metal single atoms with extremely small size were uniformly dispersed into 3D interconnected structures with open pores, it would be possible to simultaneously achieve strong absorption properties and low weight. However, the EMW absorption properties of 3D interconnected structures with open pores containing metal single atoms have been rarely investigated.

Herein, we report a general approach, namely a NaCl-templating method, to synthesize a series of 3D honeycomb-like M–N_*x*_Cs (M = Mn, Fe, Co, Cu, or Ni). Experimental results demonstrate that these 3D M–N_*x*_Cs exhibit a greatly enhanced dielectric loss compared with that of 3D NC without metal single atoms. Theoretical calculations reveal that, after the introduction of metal single atoms, new electronic states (*d*-band) are formed near the Fermi level, leading to a higher electrical conductivity of the M–N_x_C materials than of the NC matrix, thereby improving the conductive loss of 3D M–N_*x*_Cs. The charge density difference and Mulliken charges show that the interaction between the metal single atoms and adjacent N/C atoms causes charge redistribution, destructs the symmetry of the local microstructure, and then induces the formation of additional electrical dipoles, which can enhance the dipolar polarization loss of 3D M–N_*x*_Cs. As a result, our 3D M–N_*x*_Cs exhibit significantly enhanced EMW absorption properties. Typically, 3D Mn–NC with Mn–N_4_ moieties shows a minimal reflection loss (*R*_L,__min_) of –46.2 dB and an effective absorption bandwidth (EAB_10_) of 4.7 GHz when the filler ratio into the paraffin matrix is as low as 10 wt.%. These properties are superior to those of the most commonly reported EMW absorbers. Moreover, more than 1.0 g of 3D M–N_*x*_Cs can be produced in each batch through the presented strategy, suggesting the feasibility of their practical application in EMW absorption. Therefore, our findings not only systematically shed light on the relationship between M–N_x_C moieties and their dielectric behavior but also highlight a new strategy for the rational design and synthesis of lightweight EMW absorbers based on metal single atoms.

## Materials and Methods

### Preparation of the Series of 3D Honeycomb-like M–N_*x*_Cs

Typically, 0.5 g of C_8_H_11_NO_2_·HCl, 5 g of NaCl and 20 mg of a metal source (such as MnCl_2_·4H_2_O, FeCl_3_·6H_2_O, CoCl_2_·6H_2_O, CuCl_2_·2H_2_O, or NiCl_2_·6H_2_O) were dissolved into 100 mL of deionized water to form a homogeneous solution under ultrasonic processing. Subsequently, the powder was obtained after freeze-drying of the above solutions under vacuum. Next, the obtained white powder was carbonized at 800 °C with a ramp rate of 3 °C min^−1^ for 2 h in Ar atmosphere followed by washing in deionized water and acidic etching with 0.5 M H_2_SO_4_ to remove the NaCl templates and metal nanoparticles, respectively. The obtained black powder was dried at 70 °C, and the samples were named as 3D M–N_x_C.

### Preparation of the Reference Samples

For comparison, three reference samples were fabricated. The samples without metal single atoms, without the use of the NaCl templates and without the aid of the etching treatment were denoted as 3D NC, Mn–N_x_C-w, and 3D Mn NPs–NC, respectively. 3D NC was prepared under the same preparation conditions except for the fact that the metal sources were absent. Mn–N_x_C-w was prepared under the same preparation conditions except for the fact that the NaCl templates were absent. 3D Mn NPs–NC was prepared under the same preparation conditions except for the fact that acid treatment was not carried out.

### Electromagnetic Parameter Measurements

The electromagnetic parameters of the samples were measured using a vector network analyzer (Anritsu MS4644A Vectorstar) in the range of 2–18 GHz. The absorbing materials were prepared by mixing the sample powder into the paraffin matrix with a weight percentage of 10 wt.%. The mixture was then pressed into a toroidal-shaped specimen (outer diameter: 7.00 mm; inner diameter: 3.04 mm; and height: ~ 3.00 mm). Before the measurement, the electromagnetic parameter was verified using a standard Teflon sample with the same shape and size as the tested sample.

The reflection loss (*R*_L_) was calculated based on the transmission line theory according to:

1 $$Z_{in} = Z_{{\text{o}}} (\mu_{r} /\varepsilon_{r} )^{1/2} \tan \;h\left[ {j(2\pi \;fd/c)(\mu_{r} \varepsilon_{r} )^{1/2} } \right]$$

2 $$R_{L} = 20\log \left| {(Z_{in} - Z_{o} )/(Z_{in} + Z_{o}) } \right|$$

where *Z*_in_ is the input impedance, *Z*_0_ is the impedance of free space of the materials, *c* is the velocity of light in free space,* f* is the frequency of microwaves and *d* is the thickness of the absorber. *ε*_r_ (*ε*_r_ = *ε*׳ – *jε*״) is the relative complex permittivity. *µ*_r_ (*µ*_r_ = *µ*׳ − *jµ*״) is the relative complex permeability.

The attenuation constant (*α*) can be described as follows:

3 $$\alpha = \frac{\sqrt 2 \pi f}{c} \times \sqrt {(\mu^{\prime \prime } \varepsilon^{\prime \prime } - \mu^{\prime } \varepsilon^{\prime } )+\sqrt {\left( {\mu^{\prime } \varepsilon^{\prime \prime } + \mu^{\prime \prime } \varepsilon^{\prime } } \right)^{2} + (\mu^{\prime ^{\prime}} \varepsilon^{\prime \prime } - \mu^{\prime } \varepsilon^{\prime)^{2} } } }$$

### Computational Methods

The density of states (DOS) and charge density difference were calculated using the Cambridge Serial Total Energy Package (CASTEP) in Material Studio. The Perdew–Burke–Ernzerhof (PBE) in the generalized gradient approximation (GGA) form was used as the exchange–correlation function. An energy-cutoff of 400 eV for the plane-wave expansion and a 2 × 2 × 1 Monkhorst–Pack *k*-points grid was used to examine the convergence tests of the total energy with respect to the energy-cutoff and the *k*-points sampling. A vacuum space of 15 Å was selected to prevent any interaction between the adjacent periodic images of the 2D graphitized carbon. The optimized structures were calculated until the force fell below 2.0 × 10^−5^ eV Å^−1^. To evaluate the accuracy of the Mulliken charges, single-point energy calculations with a basis set cutoff of 4.4 Å were further performed. The results clearly show that the changes in the Mulliken charges are smaller than 10%, indicating that the values provided are reasonable. The dipole moment of a single-structure cell was implemented using the Dmol3 module with the GGA and PBE exchange–correlation functional.

## Results and Discussion

**Fig. 1:**
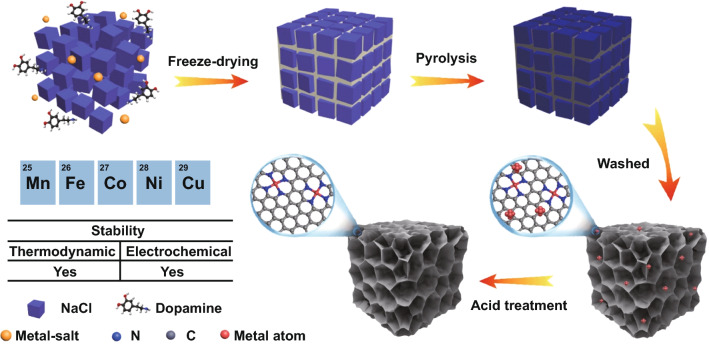
Schematic of the synthetic process for the 3D M–N_x_C structures

The synthetic process of 3D M–N_*x*_Cs structures through the NaCl-templating method is depicted in Fig. 1. NaCl, 3-hydroxytyramine hydrochloride (C_8_H_12_ClNO_2_), and metal salts (MnCl_2_·4H_2_O for Mn–N_x_C, FeCl_3_·6H_2_O for Fe–N_x_C, CoCl_2_·6H_2_O for Co–N_x_C, CuCl_2_·2H_2_O for Cu–N_x_C and NiCl_2_·6H_2_O for Ni–N_x_C) were dissolved into distilled water under stirring till a homogeneous aqueous solution was formed. The powder was then obtained after freeze-drying of the above solutions under vacuum. As the precursor solution was saturated by NaCl, the contents of dopamine and the metal source were much lower than their saturation solubility. Therefore, during the freeze-drying process, cubic NaCl crystals were firstly precipitated as 3D hard templates accompanied by sublimation of water and were subsequently coated by dopamine and the metal source. Scanning electron microscopy (SEM) and energy-dispersive X-ray spectroscopy (EDX) show that the powder after the freeze-drying process displays 3D aggregates with an irregular cubic morphology as well as C, N, and Mn elements dispersed throughout the metal salt/dopamine (organic/inorganic) coating layer on the NaCl (Fig. S1). After the dried powder was carbonized at 800 °C in an Ar atmosphere, metal ions were bonded to nitrogen atoms, thus forming the M–N_x_C structures. Due to the uniform distribution of both the metal ions and dopamine in the organic–inorganic coating layer on the NaCl, the formed M–N_x_C moieties were dispersed throughout the 3D M–N_x_C sample. Generally, isolated metal single atoms tended to aggregate and form metal nanoparticles due to their high surface energy. However, the doped N species in the carbon matrix could immobilize the metal single atoms through the strong coordination interactions between the *d* orbitals of the metal atoms and the lone pair electrons of the N atom [19, 20]. Thus, metal single atoms and nanoparticles coexisted in the 3D M–N_x_C samples. After acidic etching, the NaCl templates and the nanoparticles could be removed, leading to the formation of 3D honey-comb-like NC structures containing the uniformly dispersed metal single atoms. Notably, the freeze-drying, carbonization, and acidic etching processes were mainly involved in the synthesis strategy, and large-scale production of 3D M–N_*x*_Cs was thus easily realized. Typically, more than 1.0 g of 3D M–N_*x*_Cs could be fabricated in each batch (Fig. S2).

**Fig. 2:**
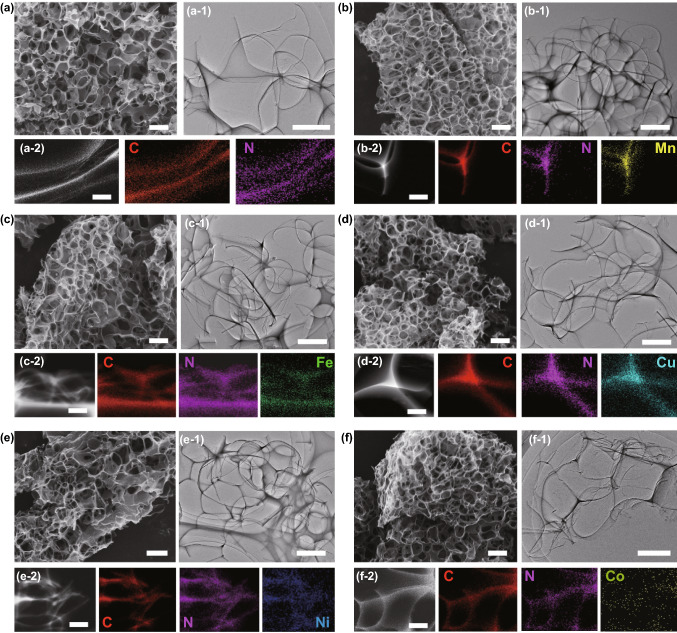
SEM images, TEM images, and EDX mapping of **a** 3D NC, **b** 3D Mn–NC, **c** 3D Fe–NC, **d** 3D Cu–NC, **e** 3D Ni–NC, and **f** 3D Co–NC. Scale bars: **a–f** 1 μm; **a1–f1** 500 nm; **a2–f2** 200 nm

The SEM images confirm that the as-prepared NC and M–N_*x*_Cs exhibit 3D honeycomb-like morphologies with interconnected open pores (Fig. 2a–f). The pore size is about 1 μm in the 3D honeycomb-like M–N_*x*_Cs, and the carbon matrix has a thickness of about 15 nm (Fig. S3). During the high-temperature carbonization process, the high thermodynamics energy can lead to the growth of NaCl into big particles, accelerating the formation of two-dimensional (2D) interpenetrating nanosheets on the surface of the big particles (Fig. 2a–f). After removal of the NaCl templates, the 3D honeycomb-like structures were formed. Such interconnected structures with open pores endow the 3D M–N_*x*_Cs with a large surface area, which facilitates the improvement of the EMW absorption performance [21]. To gain information on the specific surface area and the porosity of the 3D honeycomb-like M–N_*x*_Cs, N_2_ adsorption–desorption isotherms were measured. The adsorption–desorption isotherms of the 3D M–N_*x*_Cs exhibit type-IV loops, suggesting the presence of mesopores in these samples (Fig. S4). The pore size distribution curves indicate that the mesopores were mainly centered at about 2.3 nm (Fig. S4 and Table S1). These mesopores mainly exist on the carbon nanosheets. During the carbonization process, molten salt can flow out through the shell due to capillary action when the temperature exceeds the melting temperature of NaCl, which is attributed to the formation of mesopores [22]. The Brunauer–Emmett–Teller (BET) surface areas of the 3D M–N_*x*_Cs are in the range of 550–634.0 m^2^ g^−1^, revealing their lightweight features (Fig. S4 and Table S1). In addition, in the absence of the NaCl template, the obtained Mn–N_x_C-w sample exhibits a plate-like shape without 3D open pores (Fig. S5). This result highlights the importance of the NaCl templates for the construction of ultralight 3D honeycomb-like M–N_*x*_Cs.

**Fig. 3:**
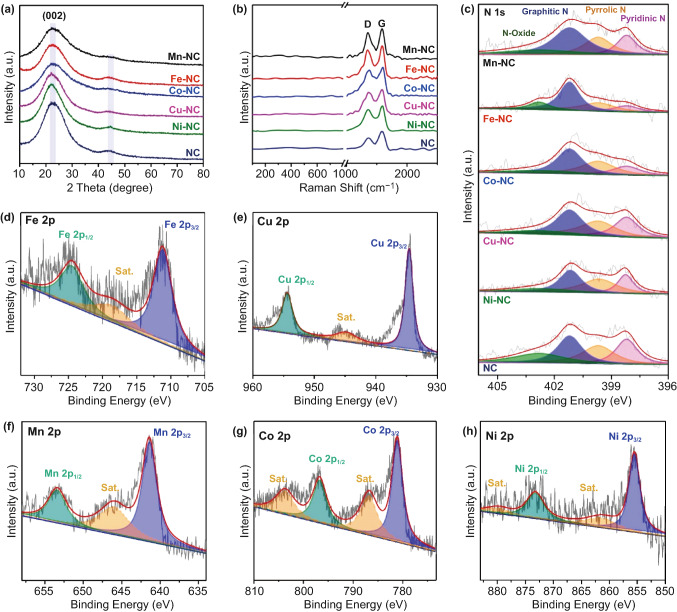
**a** XRD patterns, **b** Raman spectra, and **c** high-resolution N 1*s* spectra of the samples. Metal 2*p* spectra of **d** Fe, **e** Cu, **f** Mn, **g** Co, and **h** Ni

Figure 2a1–f1 shows transmission electron microscopy (TEM) images of the 3D honeycomb-like M–N_*x*_Cs. The TEM images show that no noticeable larger metal particles are present in the NC nanosheets. However, EDX mapping indicates that N, C, and the corresponding metals are uniformly distributed across the carbon nanosheets (Figure 2a2–f2). Thus, it can be inferred that atomically dispersed atoms exist in these 3D M–N_*x*_Cs. Notably, the high-resolution TEM (HRTEM) images of the 3D M–N_*x*_Cs show that mesopores with a size of approximately 2.3 nm (highlighted by yellow circles) are distributed in the NC nanosheets (Fig. S6), which is consistent with the N_2_ adsorption–desorption isotherm measurements. To further analyze the composition and structure of the 3D M–N_*x*_Cs, X-ray diffraction (XRD), Raman spectroscopy, and X-ray photoelectron spectroscopy (XPS) measurements were performed. Two broad diffraction peaks centered at 24° and 43° are apparent in the XRD patterns of the 3D M–N_*x*_Cs (Fig. 3a), which are assigned to the reflections of the NC nanosheets [18]. Diffraction peaks from the metal nanoparticles or metallic compounds are not detected in the XRD patterns, which is due to the low metal content in 3D M–N_*x*_Cs. Figure 3b shows the Raman spectra of the 3D M–N_*x*_Cs, in which the two peaks centered at 1349 and 1583 cm^−1^ correspond to the D and G bands of the carbon materials, respectively. The intensity ratios of the D band to the G band (*I*_D_/*I*_G_) for the 3D M–N_*x*_Cs (0.972 for Mn–N_x_C, 0.965 for Fe–N_x_C, 0.973 for Cu–N_x_C, 0.961 for Ni–N_x_C, and 0.969 for Co–N_x_C) are larger than that of 3D NC (0.940), indicating that more defects are produced upon the introduction of metal single atoms into the NC nanosheets [19]. The Raman scattering peaks of the metal nanoparticles or metallic compounds are not detected (100–800 cm^−1^), which may also be due to the low metal content in 3D M–N_*x*_Cs. The XPS survey spectra indicate that C, N, O, and the corresponding metal elements are present in the 3D M–N_*x*_Cs, in line with the EDX element mappings (Fig. S7). The three peaks centered at 284.7, 285.3, and 288.8 eV in the XPS spectra of C 1*s* (Fig. S8) for all samples are ascribed to C–C, C–N, and O–C=O, respectively. The XPS spectra of N 1*s* (Fig. 3c) can be deconvoluted into four types of nitrogen species: pyridinic-N (398.2 eV), pyrrolic-N (399.7 eV), graphitic-N (401.2 eV), and N-oxide (402.8 eV) [23]. According to the XPS results, the content of the nitrogen species in 3D M–N_*x*_Cs is larger than that in 3D NC (Table S2), suggesting that the presence of metal atoms can increase the nitrogen content in the carbon framework through the formation of metal–N_x_ bonds [19]. High-resolution 2*p* XPS spectra of the metal species indicate the coexistence of the Fe^2+^ and Fe^3+^ species in Fe–N_x_C (Fig. 3d) and the coexistence of the Cu^+^ and Cu^2+^ species in Cu–N_x_C (Fig. 3e) [24]. The valence states of the Mn, Co, and Ni species in Mn–N_x_C, Co–N_x_Cs, and Ni–N_x_Cs are + 2 (Fig. 3f–h) [25–29]. Notably, the signals of the metal ^(0)^ states are not detected in the XPS spectra of all 3D M–N_*x*_Cs. Based on the XRD, TEM, XPS, Raman, and EDX results, it can be concluded that the metal species in our 3D M–N_*x*_Cs are isolated metal atoms. The content of the metal single atoms in the 3D M–N_*x*_Cs determined using inductively coupled plasma optical emission spectrometry (ICP-OES) is in the range of 1.12–1.80 wt.% (Table S3).

**Fig. 4:**
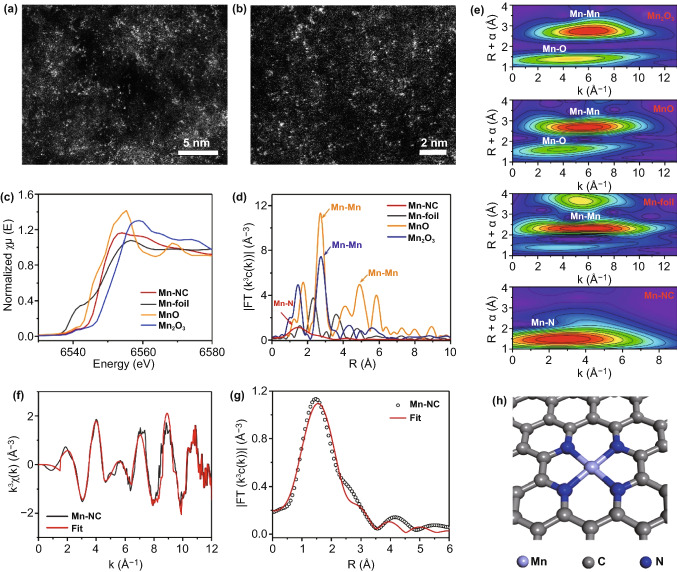
**a–b** AC HAADF-STEM images of 3D Mn–NC. Isolated single Mn atoms are highlighted by yellow circles. **c** Normalized XANES spectra at the Mn K-edge of the Mn foil, MnO, Mn_2_O_3_, and Mn–NC. **d** FT-EXAFS spectra of the Mn foil, MnO, Mn_2_O_3_, and Mn–NC. **e** WT of the Mn foil, MnO, Mn_2_O_3_, and Mn–NC. **f** EXAFS fitting curve for Mn–NC in the *k*-space. **g** EXAFS fitting curve for Mn–NC in the *R*-space. **h** Configuration of Mn–N_4_ in 3D Mn–NC

Among the various 3D M–N_*x*_Cs, 3D Mn–N_x_C exhibits the best EMW absorption properties. Thus, the microstructure of Mn single atoms in 3D Mn–N_x_C is predominantly analyzed at the atomic level via aberration-corrected high-angle annular dark-field SEM (AC HAADF-STEM), as shown in Fig. 4a, b. The bright dots correspond to heavier Mn atoms with a higher atomic number, which are uniformly distributed across the carbon matrix. To further analyze the coordination environment of the Mn single atoms, X-ray absorption fine structure (XAFS) measurements were carried out. Figure 4c shows Mn K-edge X-ray absorption near-edge structure (XANES) spectra of 3D Mn–NC, Mn foil, MnO, and Mn_2_O_3_. The XANES curves of 3D Mn–NC are located between MnO and Mn_2_O_3_, but are closer to those of MnO, suggesting that the oxidation state of the Mn species in 3D Mn–NC is close to + 2, in good agreement with the XPS analysis [25]. The Fourier transform (FT) of the k^3^-weighted extended X-ray absorption fine structure (EXAFS) spectra of 3D Mn–NC in Fig. 4d displays one main peak at about 1.5 Å, which is assigned to the Mn–N bonds. The peaks at about 2.6 and 4.8 Å, which correspond to the Mn–Mn bonds, are observed in the EXAFS spectra of the Mn foil, MnO, and Mn_2_O_3_, while are not found in the EXAFS spectra of 3D Mn–NC, suggesting that metallic Mn and Mn oxides are not present in 3D Mn–NC. To gain more information about the atomic configuration of the Mn species in 3D Mn–NC, the wavelet transform (WT)-EXAFS analysis was performed as it can provide higher sensitivity in both the *R*- and *k*-space. As illustrated in Fig. 4e, the WT signals assigned to the Mn–Mn and Mn–O bonds are not detected in 3D Mn–NC, which is in contrast with the case of the WT-EXAFS plots of the Mn foil, MnO, and Mn_2_O_3_. Furthermore, the WT contour plots of 3D Mn–NC only display one intensity maximum at about 2.8 Å, corresponding to the Mn–N coordination. Based on the AC HAADF-STEM, XAFS, and WT-EXAFS analyses, it can be inferred that the Mn atoms atomically dispersed on the N-doped carbon matrix are bonded to the N atoms. EXAFS fitting was performed to extract the structure parameters and obtain the quantitative chemical configuration of the Mn atoms (Fig. 4f, g and Table S4). The fitting results show that the coordination number of the Mn single atoms in 3D Mn–NC is four, with the mean bond length of Mn–N being 2.16 Å (Table S4). The analyses above demonstrate that each Mn atom in 3D Mn–N_x_C is coordinated by four N atoms, as shown in Fig. 4h.

**Fig. 5:**
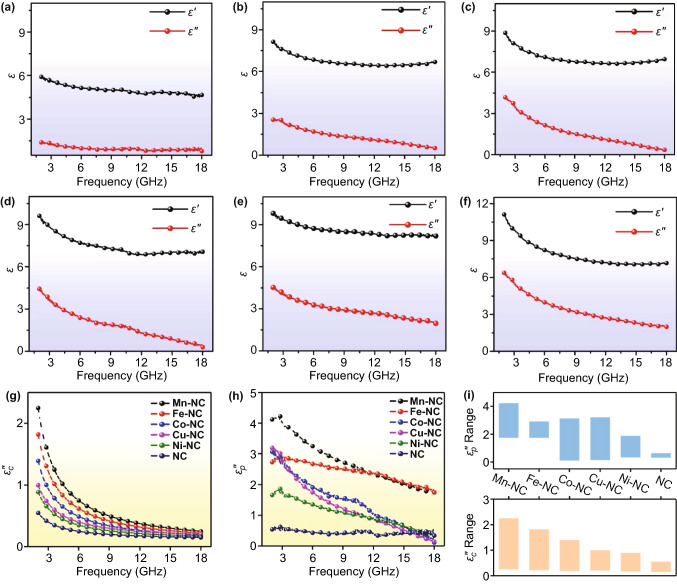
Frequency dependence of *ε*_r_. **a** 3D NC, **b** 3D Ni–NC, **c** 3D Cu–NC, **d** 3D Co–NC, **e** 3D Fe-NC, and **f** 3D Mn–NC. **g–h** Frequency dependence of *ε*_c_״ and *ε*_p_״ of 3D M–NCs and 3D NC. **i** The *ε*_c_״ and *ε*_p_״ values of 3D M–NCs and 3D NC cover the range of 2–18 GHz

These structural characterizations indicate that our as-prepared M–N_*x*_Cs have 3D interconnected porous morphologies, large surface areas, and abundant polarization centers, such as metal single atoms coordinated with N atoms, which can synergistically improve their EMW absorption performance [30, 31]. To evaluate the EMW absorption properties of the 3D M–N_*x*_Cs, the electromagnetic parameters, including the relative complex permittivity (*ε*_r_ = *ε*׳ – *jε*״) and permeability (*µ*_r_ = *µ*׳ − *jµ*״) were measured. The filler ratio of the 3D M–N_*x*_Cs samples in the paraffin wax was controlled to be 10 wt.%. The real (*µ*׳) and imaginary (*µ*״) parts of *µ*_r_ for all the 3D M–N_*x*_Cs samples and the 3D NC matrix were measured almost 1 and 0, respectively, which is due to the nonmagnetic properties of these samples (Fig. S9). Thus, the 3D M–N_*x*_Cs with atomically dispersed metal atoms have a negligible magnetic loss toward EMW, which is also confirmed by their low magnetic loss tangent (tan *δ*_µ_ = *µ*״/*µ*׳) (Fig. S9). Figure 5a–f compares the real part (*ε*׳) of *ε*_r_ of the 3D M–N_*x*_Cs and 3D NC. It can be observed that the *ε*׳ value of 3D NC decreases from 5.8 to 4.5 with an increase in the frequency from 2 to 18 GHz. Although the *ε*׳ values of the 3D M–N_*x*_Cs also decrease, they remain much higher than that of 3D NC. For example, the minimal *ε*׳ values of 3D Ni–NC, Cu–NC, Co–NC, Fe–NC, and Mn–NC in the range of 2–18 GHz are 6.6, 6.9, 7.0, 8.2, and 7.1, respectively. The increased *ε*׳ values of the 3D M–N_*x*_Cs suggest that they have a higher capacity to store EMW energy [32]. Figure 5a–f shows the changes in the imaginary part (*ε*״) of *ε*_r_ for the 3D M–N_*x*_Cs and 3D NC as the frequency increases. Clearly, the 3D M–N_*x*_Cs have a much higher *ε*״ value than 3D NC. Typically, the *ε*״ value of 3D NC is in the range of 1.4–0.7. By contrast, the range of the *ε*״ value increases to 2.7–0.5 for 3D Ni–NC, 4.0–0.3 for 3D Cu–NC, 4.3–0.2 for 3D Co–NC, 4.5–2.0 for 3D Fe–NC, and 6.3–1.9 for 3D Mn–NC. These results indicate that the 3D M–N_*x*_Cs have also a stronger capacity to dissipate EMW energy compared with 3D NC, which is confirmed by their higher dielectric loss tangent (tan *δ*_ε_ = *ε*״/*ε*׳) (Fig. S9). To uncover the reasons for the increased dielectric loss of the 3D M–N_*x*_Cs, Cole–Cole plots are illustrated in Fig. S10. It can be observed that the dielectric loss of the 3D M–N_*x*_Cs and 3D NC is mainly related to the conductive loss (the straight line in the plots) and the polarization relaxation loss (the semicircles in the plots) [33]. To determine the conductive loss (*ε*_c_״ = *σ*/2π*fε*_0_, where *σ*, *f*, and *ε*_0_ are the electrical conductivity, frequency of the electromagnetic wave, and vacuum permittivity, respectively), the electrical conductivity of the absorbers based on the 3D M–N_*x*_Cs and 3D NC was measured using a Hall-effect system. The measured results indicate that the conductivities of 3D Mn–NC, Fe–NC, Co–NC, Cu–NC, and Ni–NC are 4.40, 4.16, 4.02, 3.96, and 3.87 S m^−1^, respectively, thus much higher than that of 3D NC (1.01 S m^−1^) (Table S5). As shown in Fig. 5g, the calculated *ε*_c_״ value of 3D NC decreases from 0.52 to 0.12. By contrast, the *ε*_c_״ value is in the range of 2.7–0.22 for 3D Mn–NC, 1.82–0.22 for 3D Fe–NC, 1.39–0.19 for 3D Co–NC, 1.0–0.2 for 3D Cu–NC, and 0.89–0.17 for 3D Ni–NC, thus much higher than that of 3D NC. Thus, the introduction of metal single atoms into the 3D NC matrix can remarkably improve the conductivity and, correspondingly, the conductive loss [33]. Based on the *ε*_c_״ values, the polarization relaxation loss (*ε*_p_״ = *ε*״ – *ε*_c_״) of the 3D M–N_*x*_Cs and 3D NC can be extracted [34]. As shown in Fig. 5h, the *ε*_p_״ values of the 3D M–N_*x*_Cs samples are also higher than those of 3D NC, indicating that the introduction of metal single atoms into the 3D NC matrix increases the dipole polarization loss. According to these analyses, it can be concluded that both the increased conductivity and polarization are responsible for the increased dielectric loss of the 3D M–N_*x*_Cs. Notably, the conductive and polarization relaxation losses of our 3D M–N_*x*_Cs follow the order 3D NC < 3D Ni–NC < 3D Cu–NC < 3D Co–NC < 3D Fe–NC < 3D Mn–NC (Fig. 5i), suggesting that Mn single atoms are the most beneficial for the enhancement of the dielectric loss.

**Fig. 6:**
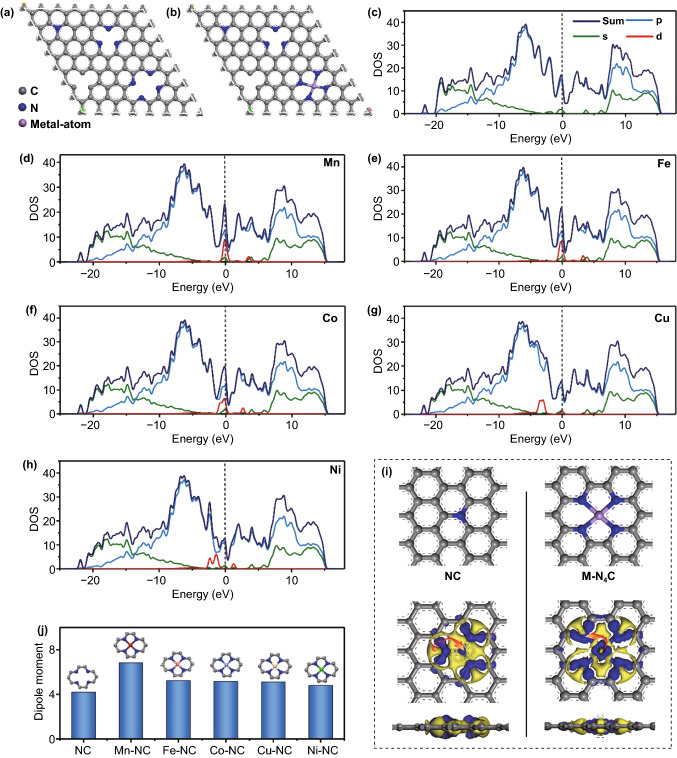
Schematic structural models of **a** NC and **b** M–N_4_C structures. **c–h** Calculated DOS of the NC and M–N_4_C structures. **i** Calculated charge density difference of the NC and Mn–N_4_C structures. **j** Calculated dipole moment of the NC and M–N_4_C structures (M = Mn, Fe, Co, Cu, or Ni)

To provide an in-depth understanding of the influence of the configuration of metal single atoms on the dielectric loss, density functional theory (DFT) calculations of the 3D M–N_*x*_Cs were carried out. Considering the M–N_4_C moieties have superior thermodynamic and electrochemical stability on the carbon matrix with respect to other configurations, the M–N_4_C models were adopted [35]. Figure 6a, b shows the calculated models for the NC matrix and M–N_4_C, respectively. The density of states (DOS) was first calculated to elucidate the change in the electronic structure of the M–N_4_Cs upon the introduction of metal single atoms. As shown in Fig. 6c–h, after the introduction of metal single atoms, new electronic states (*d*-band) are formed near the Fermi level, leading to the M–N_4_Cs samples having a higher electrical conductivity than the NC matrix [36]. Since the Mn, Fe, Co, Cu, and Ni transition metals are located in the same periodic group in the periodic table of the elements, they have similar fully occupied *s* and *p* orbitals. Thus, the increase in the DOS of the *d* orbitals near the Fermi level is the main reason for the enhancement in the conductivity of the 3D M–N_4_C samples. The improvement in the conductivity can contribute to the conductive loss, resulting in an enhancement in the dielectric loss properties. Additionally, according to the DOS calculations, the conductivity of the M–N_4_Cs samples decreases as follows: Mn–N_4_C > Fe–N_4_C > Co–N_4_C > Cu–N_4_C > Ni–N_4_C. The order of the conductivity of the M–N_4_Cs is relative to their electronegativity (Mn < Fe < Co < Cu < Ni). The highest electrical conductivity of Mn–N_4_C results in its largest conductive loss, which is consistent with the results of Fig. 5g. In order to visualize the interaction between the metal single atoms and adjacent N and C atoms, the charge density difference and Mulliken charges were calculated. As shown in Figs. 6i and S11, the introduction of metal single atoms of M–N_4_Cs leads to charge redistribution, which destructs the symmetry of the local microstructure and induces the formation of additional electric dipoles. These electric dipoles oscillate under the applied external electromagnetic field, which is beneficial to increase the dipolar polarization loss. 3D Mn–N_4_C has the highest metal content and nitrogen species dopants among the 3D samples, leading to its largest dipolar polarization loss. The calculated Mulliken charges reveal changes in the metal atoms, N atoms, and C atoms in the charge density (Table S6). Typically, the Mulliken charges of N and C atoms in the NC matrix are –0.33 and 0.18 in the local NC structure, respectively. In Mn–NC, the Mulliken charges of the Mn, N, and C atoms are 1.17, –0.43, and 0.11, respectively. Since the N and C atoms possess a higher electronegativity than the metal atoms, electrons are more easily attracted by the N and C atoms in M–N_4_Cs, leading to an abundance of electrons in N and C. Thus, the M–N_4_Cs have a larger electric dipole moment than the NC matrix, which can boost their dipolar polarization loss. To provide in-depth insights into the polarization ability of the M–N_4_Cs, their dipole moments were calculated [37]. As shown in Fig. 6j, the calculated dipole moments of the M–N_4_Cs are larger than those of the NC matrix, further confirming that the introduction of metal single atoms can increase the polarization loss of NC. Notably, Mn–N_4_C shows the highest dipole moment among the M–N_4_Cs, which is consistent with the experimentally measured polarization relaxation loss (Fig. 5h). In addition, the pyrrolic-N and pyridinic-N species are beneficial for the dipolar relaxation loss, whereas the graphitic N species are essential for enhancing the conduction loss [23]. The Cu–N_4_C and Ni–N_4_C samples have an amount of N dopants close to that of the NC; however, they have much high dielectric loss values, suggesting that metal single atoms play a dominant role in the improvement of the dielectric loss properties of 3D M–N_4_Cs.

**Fig. 7:**
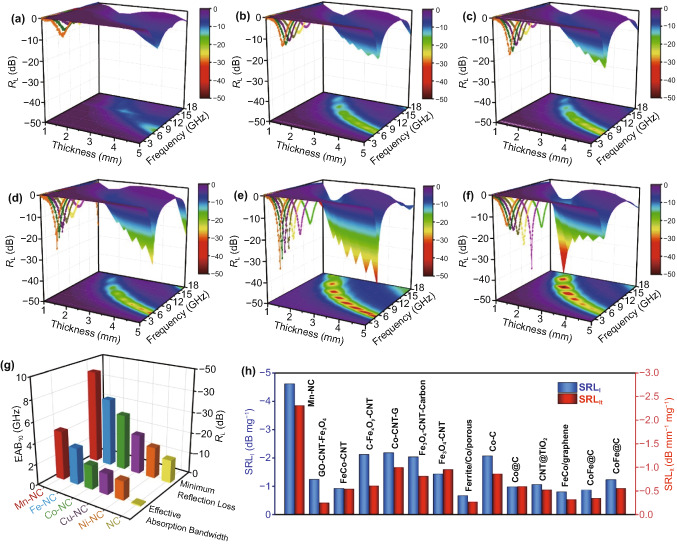
Reflection loss curves and 3D plots of **a** 3D NC, **b** 3D Ni–NC, **c** 3D Cu–NC, **d** 3D Co–NC, **e** 3D Fe–NC, and **f** 3D Mn–NC. **g** Comparison of *R*_L, min_ and EAB_10_ for 3D M–NCs and 3D NC. **h** *SRL*_l_ and *SRL*_lt_ values of 3D Mn–NC with the reported carbon-based absorbers

Both the experimental results and the theoretical calculations confirm that the introduction of Mn, Fe, Co, Cu, or Ni metal single atoms into the NC structure can improve the conductive and polarization losses. In particular, 3D Mn–NC exhibits the highest conductive and polarization losses (Fig. 5i) and is thus expected to have optimal EMW absorption properties. The EMW absorption performance of the 3D M–N_*x*_Cs and 3D NC were evaluated in terms of the reflection loss (*R*_L_) based on the transmission line theory [38, 39]. Figure 7a–f shows the *R*_L_–*f* curves and the corresponding 3D plots of the 3D M–N_*x*_Cs and 3D NC with a thickness of 1.0–5.0 mm over the 2–18 GHz range. The minimal *R*_L_ (*R*_L, min_) value of 3D NC is –11.2 dB, and the effective absorption bandwidth (EAB_10_) is only 0.2 GHz at a thickness of 5.0 mm. According to the *R*_L, min_ and EAB_10_ values along with the matching thickness, the EMW absorption properties of the 3D M–N_*x*_Cs are improved significantly, as summarized in Fig. 7g. In particular, the *R*_L, min_ and EAB_10_ values for 3D Mn–NC can reach –46.2 dB and 4.7 GHz, respectively. Furthermore, even though the thickness is reduced to 1.7 mm, 3D Mn–NC still exhibits superior EMW absorption properties with an *R*_L, min_ value of –20 dB (Fig. S12). Notably, the EMW absorption properties of the 3D M–N_*x*_Cs follow the order 3D NC < 3D Ni–NC < 3D Cu–NC < 3D Co–NC < 3D Fe–NC < 3D Mn–NC (Fig. 7g), which is consistent with the order of the dielectric loss values. Therefore, the improved EMW absorption performance of the 3D M–N_*x*_Cs samples is attributed to the increase in both conductive loss and polarization loss compared with those of 3D NC. As discussed above, the introduction of metal single atoms increased the conductive loss and polarization loss of the 3D M–N_*x*_Cs samples. Thus, the enhanced EMW absorption properties of the 3D M–N_*x*_Cs samples are attributed to the presence of metal single atoms in these samples.

In addition, the degree of impedance matching (*M*_z_) and the attenuation constant (*α*) can be used to evaluate the performance of the EMW absorption materials. Generally, *M*_z_ with a value close to 1 indicates that the incident EMW can almost enter the interior of the absorber without reflection at the surface, which can endow the absorber with a stronger EMW absorption performance [40]. Fig. S13 shows the *M*_z_–*f* plots of the 3D M–N_*x*_Cs and 3D NC over the 2–18 GHz range. The *M*_z_ values of the 3D M–N_*x*_Cs are closer to 1 than that of 3D NC, suggesting that the 3D M–N_*x*_Cs have better impedance matching characteristics than 3D NC. Under impedance matching conditions, the larger the attenuation constant of an absorber is, the better its EMW attenuation capacity will be [41]. As shown in Fig. S14, *α* is in the range of 72.1–239.7 for 3D Mn–NC, 35.6–231.0 for 3D Fe–NC, 45.7–105.2 for 3D Co–NC, 47.2–102.9 for 3D Cu–NC, and 34.9–98.2 for 3D Ni–NC, thus larger than that of 3D NC (17.9–88.2), indicating that the 3D M–N_*x*_Cs have an enhanced attenuation capacity toward the EMW propagated through the absorbers. Thus, the 3D M–N_*x*_Cs not only exhibit better EMW absorption performance but also higher attenuation capacity than 3D NC. In addition, the EMW absorption properties of 3D Mn–NC were compared with those of other typical carbon-based EMW absorbers. Two specific values, namely *SRL*_l_ (*SRL*_l_ = *R*_L_/filler loading) and *SRL*_lt_ (*SRL*_lt_ = *R*_L_/(thickness × filler loading)) are more appropriate indicators to evaluate the EMW absorption efficiency of different absorbers [42]. The |*SRL*_l_| and |*SRL*_lt_| values of 3D Mn–NC can reach 4.62 dB mg^−1^ and 2.31 dB mm^−1^ mg^−1^ (assume that the mass of each absorber is 100 mg), respectively, which are much higher than those of previously reported EMW absorbers (Fig. 7h and Table S7). Thus, 3D Mn–NC is a potential candidate for highly efficient, ultrathin, and lightweight EMW absorbers.

The results above demonstrate that the 3D Mn-NC sample containing metal single atoms exhibits excellent EMW absorption properties. To highlight the important effect of the Mn single atoms on the absorption properties, the electromagnetic properties of 3D Mn NPs–NC were investigated in detail. The low-magnified SEM image shows that the sample exhibits a 3D honeycomb-like morphology (Fig. S15a), but the high-magnified SEM image reveals that some nanoparticles are anchored on the N-doped carbon sheets (Fig. S15b). Figure S16 shows that the 3D Mn NPs–NC sample has a weak magnetic loss toward EMW. As shown in Fig. S17a, b, the *ε*׳ and *ε*״ values of 3D Mn NPs–NC are higher than those of 3D NC, indicating that the 3D Mn NPs–NC sample has stronger storage and dissipation abilities toward electromagnetic energy than 3D NC. The measured conductivity of 3D Mn NPs–NC is 7.68 S m^−1^, which is larger than that of 3D NC. Thus, the increased dielectric loss of the 3D Mn NPs–NC sample compared with that of 3D NC is partially attributed to its increased conductive loss (Fig. S17c). Apart from conduction loss, the polarization loss of 3D Mn NPs–NC is also higher than that of 3D NC, as shown in Fig. S17d. Thus, the introduction of nanoparticles into the 3D NC matrix can also result in an enhancement of its dielectric loss. However, the dielectric loss values of the 3D Mn NPs–NC sample are lower than those of the 3D Mn–NC sample. This indicates that the presence of nanoparticles in the sample limits the increase in the dielectric loss, which may be due to their different specific surface areas. Fig. S18 shows the N_2_ adsorption–desorption isotherms of the 3D Mn NPs–NC sample. The BET surface area of the 3D Mn NPs–NC sample was calculated to be 508 m^2^ g^−1^, which is lower than that of 3D Mn–NC. This means that the volume ratio of 3D Mn–NC in the paraffin matrix is larger than that of 3D Mn NPs–NC. On the one hand, the higher volume ratio makes the conductive network more form in the absorber, facilitating the increase in conduction loss. On the other hand, the higher volume ratio endows the absorber with a larger number of single-atom polarization centers, leading to a higher polarization loss [43]. Figure S19 shows that the *R*_L, min_ and EAB_10_ values of 3D Mn NPs–NC are –35.4 dB and 2.9 GHz, respectively. The EMW absorption performance of 3D Mn NPs–NC is inferior to that of 3D Mn–NC, but superior to that of 3D NC, which is consistent with the order of their dielectric loss. In addition, the Mn–N_x_C-w sample shows inferior EMW absorption properties compared to 3D Mn-NC, indicating that the 3D porous structure has a positive effect on the absorption performance (Fig. S20). However, the EMW absorption properties of Mn–N_x_C-w outperform those of 3D NC (Figs. S20 and 7a). Therefore, the metal single atoms in 3D M-N_x_C play a key role in its EMW absorption properties, while the 3D porous structure has a secondary effect. The EMW absorption properties of the 3D Mn–NC sample with different filler ratios were also investigated. As shown in Fig. S21a, the *R*_L, min_ and EAB_10_ values for 3D Mn–NC with a filler ratio of 5 wt.% are –17.5 dB and 3.3 GHz, respectively. When the filler ratio is increased to 15 wt.%, the *R*_L, min_ and EAB_10_ values of 3D Mn–NC are –35.3 dB and 4.2 GHz, respectively (Fig. S21b). Therefore, the optimal filling ratio of 3D Mn–NC in the paraffin matrix is 10 wt.% in this work.

**Fig. 8:**
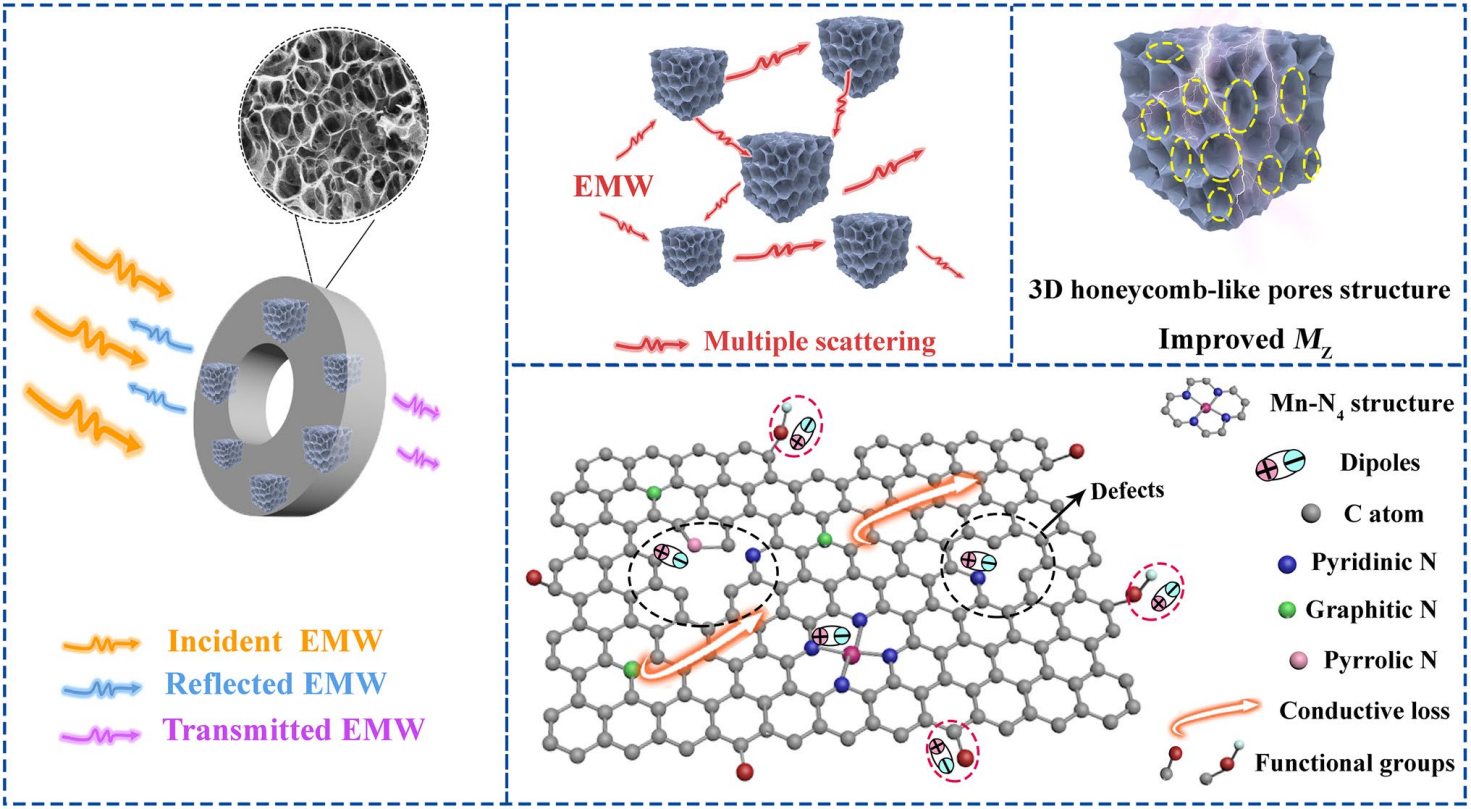
Schematic illustration of the EMW absorption mechanism of 3D Mn–NC

Based on the above discussion, the EMW absorption mechanism of the 3D honeycomb-like M–N_x_C is illustrated in Fig. 8. Firstly, the introduction of metal single atoms can increase the DOS of the *d* orbitals near the Fermi level, resulting in an enhancement in conductive loss property. Secondly, M–N_4_C moieties, surface functional groups (such as N dopants, C-O), and defects can induce the formation of electric dipoles, which are beneficial to increase the dipolar polarization loss [44–46]. Thirdly, the presence of numerous pores in the 3D M–N_x_C structures can improve their surface area and impedance matching characteristics as well as reduce the filler ratio in the paraffin matrix [47]. Besides, these unique 3D interconnected honeycomb-like structures can cause multiple scattering, which can enhance the absorption of incident EMW [48, 49].

The stability is also important for the practical application of 3D Mn–NC [50]. Firstly, the 3D Mn–NC sample was fabricated through a strong acid etching process, and thus it is characterized by good acid corrosion resistance. To further investigate the stability, the structural characterizations were carried out for the 3D Mn–NC powder that was stored in an ambient environment for three months. The XRD, ICP, SEM, and TEM measurements indicate that the phase composition, content of the metal single atoms, and microstructure of the stored powder have negligible changes compared to the sample fabricated freshly (Figs. S22-S24). The same is true for the relative complex permittivity (Fig. S25). The good stability and the strong absorption properties of 3D Mn–NC suggest that this sample is a promising candidate for EMW absorption. Overall, our study demonstrates that anchoring atomically dispersed metal atoms into carbon nanomaterials is an efficient strategy for constructing high-performance EMW absorbers.

## Conclusion

In summary, 3D honeycomb-like M–N_*x*_Cs (M = Mn, Fe, Co, Cu, or Ni) with large surface area and abundant pores, including mesopores with a size of about 2.3 nm and macropores with a size of about 1 μm, were successfully prepared through the NaCl-templating method. The uniformly dispersed metal single atoms in the 3D M–N_*x*_Cs were identified via XRD, TEM, XPS, Raman, EDX, and AC HAADF-STEM investigations. Experimental results indicate that the 3D M–N_*x*_Cs exhibit a greatly enhanced dielectric loss compared with the 3D NC matrix. Theoretical calculations demonstrate that the increase in the DOS of the *d* orbitals near the Fermi level improves the electrical conductivity, thereby enhancing the conductive loss of the 3D M–N_*x*_Cs. The calculations of the charge density difference and Mulliken charges indicate that the interaction between the metal single atoms and adjacent N/C atoms leads to a charge redistribution, destructs the symmetry of the local microstructure, and then induces the formation of additional electrical dipoles, which boost the dipolar polarization loss of the 3D M–N_*x*_Cs. Consequently, these 3D M–N_*x*_Cs exhibit significantly enhanced EMW absorption properties compared with 3D NC without metal single atoms. Particularly, the |*SRL*_l_| and |*SRL*_lt_| values of 3D Mn–NC are thus much higher than those of the most commonly reported EMW absorbers. Furthermore, more than 1.0 g of 3D M–N_*x*_Cs can be fabricated in each batch, suggesting their practical application in the large-scale production of EMW absorbers. Our insights into the EMW absorption mechanism of metal single atoms open up new opportunities for the rational design of lightweight, highly efficient EMW absorbers based on metal single atoms.

## Supplementary Information

Below is the link to the electronic supplementary material.

Supplementary file1 (DOCX 4102 KB)
