# Supplementary material for: Identification of the Intrinsic Dielectric Properties of Metal Single Atoms for Electromagnetic Wave Absorption
Source: Nanomicro Lett. 2021 Dec 11;14:27. doi: 10.1007/s40820-021-00773-6 (PMC8665961; doi:10.1007/s40820-021-00773-6)
Supplement: Supplementary file 1 — Supplementary file1 (DOCX 4102 KB) [file 40820_2021_773_MOESM1_ESM.docx]

Supporting Information

**Identification of the Intrinsic Dielectric Properties of Metal Single Atoms for Electromagnetic Wave Absorption**

Xinci Zhang^1^, Yanan Shi^1^, Jia Xu^1^, Qiuyun Ouyang^1^, Xiao Zhang^1,*^, Chunling Zhu^2,*^, Xiaoli Zhang^3^ and Yujin Chen^1, 2, 3*^

^1^ Key Laboratory of In-Fiber Integrated Optics, College of Physics and Optoelectronic Engineering, Harbin Engineering University, Harbin 150001, China

^2^ College of Materials Science and Chemical Engineering, Harbin Engineering University, Harbin 150001, China

^3^ School of Materials Science and Engineering, Zhengzhou University, Zhengzhou 450001, China

* Corresponding authors.

E-mail: zhangxiaochn@hrbeu.edu.cn; zhuchunling@hrbeu.edu.cn; chenyujin@hrbeu.edu.cn

**Material c**haracterization:

The morphology and size of the synthesized samples were characterized via XRD using an X’Pert Pro diffractometer with Cu K*α* radiation (*λ* = 1.5418 Å). The samples were scanned from 10° to 70° at a scanning rate of 5°/min. Raman spectroscopy was conducted to characterize the extent of disorder in the carbon materials using a Lab RAM Aramis micro Raman spectrometer with an excitation wavelength of 488 nm and a spot size of 2 µm. The morphology of all samples was observed using a Hitachi SU8000 scanning electron microscope at an accelerating voltage of 5–20 kV. The samples were pasted onto conductive tapes for SEM observations. TEM images were acquired using a JEM-2100 transmission electron microscope operating at a voltage of 200 kV. SEM-EDX analyses were performed to confirm the elemental contents of the samples. The ethanol solution containing the sample was treated using ultrasounds for 5 min, and the solution was then dripped onto a copper grid for TEM observations. XPS measurements were carried out using an X-ray photoelectron spectrometer (K-Alpha, Thermofisher Scientific Company) with Al K*α* radiation generated at 12 kV and 150 W. The binding energies of all samples were determined using the carbon C 1*s* peak (284.6 eV) as a reference. A Micromeritics ASAP 2010 micropore size analyzer was used to measure the specific surface area of the sample from the linear portion of the BET plots (*P*/*P*_0_ = 0.01–0.10) at 77 K. Approximately 0.2 g of catalyst was placed in a quartz tube. ICP-OES measurements were performed to determine the metal contents in the catalysts. The conductivity of the samples was determined using an X3 Hall Effect Test System (Semishare International Limited).

**XAFS measurements:**

The obtained XAFS data was processed using the Athena software (version 0.9.26) for background, pre-edge line, and post-edge line calibrations. Then, FT fitting was carried out with the Artemis software (version 0.9.26). A *k*^3^ weighting, a *k*-range of 2–5 Å^−1^, and an *R*-range of 1–4 Å were used for the fitting of the Mn sample. The coordination number, bond length, Debye–Waller factor, and *E*_0_ shift (*CN*, *R*, *σ*^2^, Δ*E*_0_) were fitted without being fixed, constrained, or correlated. For the WT analysis, the *χ*(*k*) value exported from Athena was imported into the Hama Fortran code. The parameters were as follows: *R*-range, 1–4 Å; *k*-range, ~0–13 Å^−1^ for standers (0–6Å^−1^ for the Mn sample); *k*-weight, 2. The Morlet function with *κ* = 10 and *σ* = 1 was used as the mother wavelet to provide the overall distribution.

**Figures and Tables**


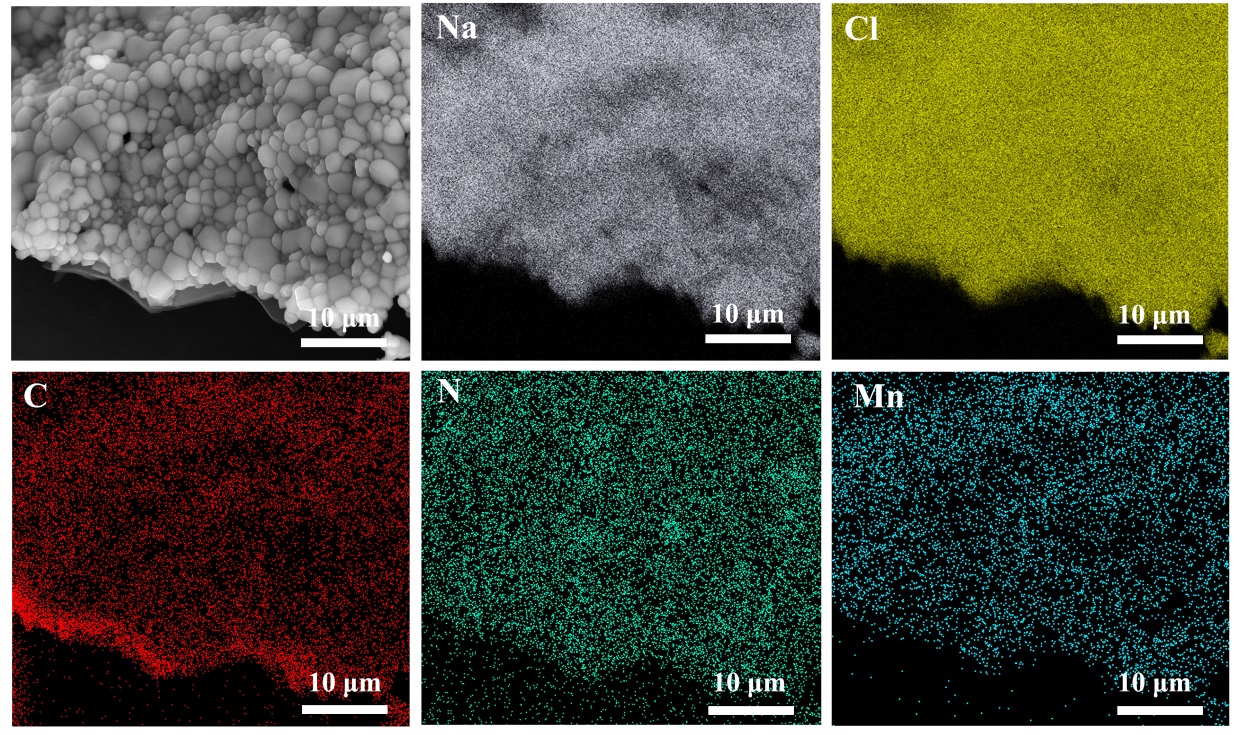


**Fig. S1** SEM image and EDX mapping of the 3D Mn–NC precursor after freeze-drying process.


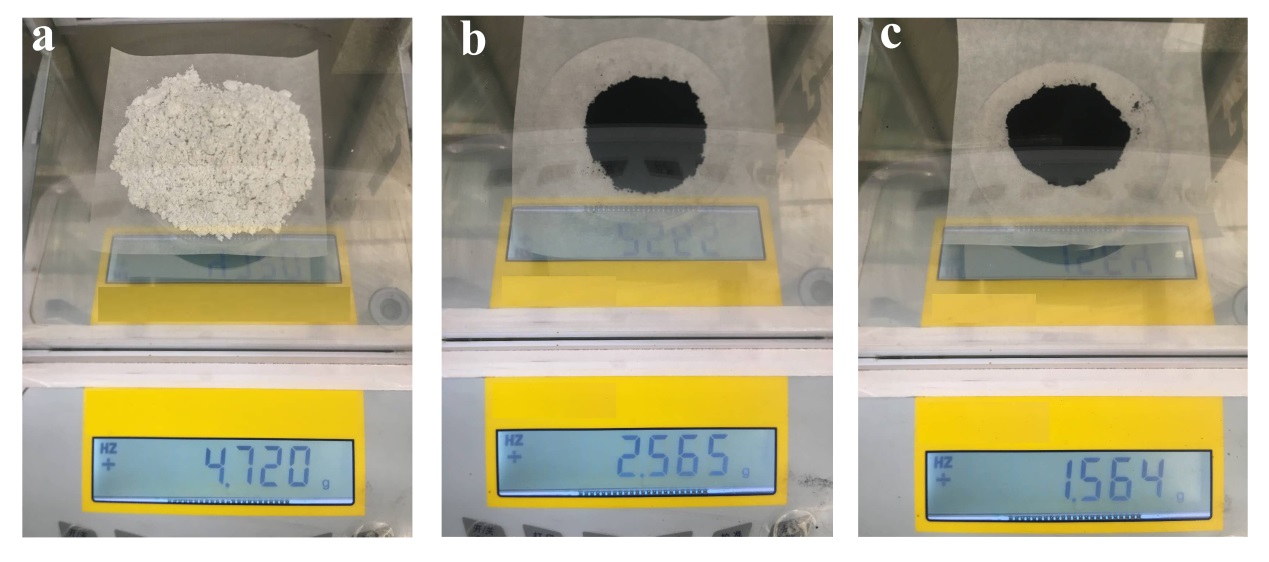


**Fig. S2** Photos of 3D Mn–NC production in each batch. **a** The precursor after freeze-drying process. **b** The sample after carbonized at 800°C. **c** The finally product of 3D Mn–NC.


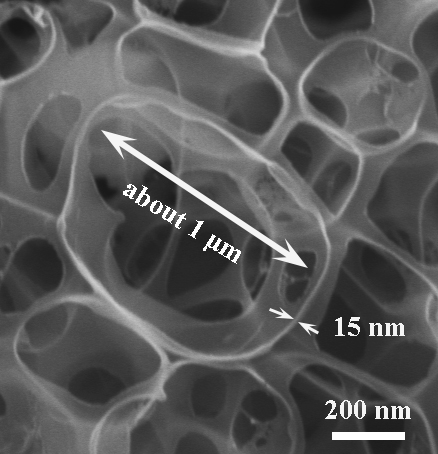


**Fig. S3** SEM images of the 3D Mn–NC.


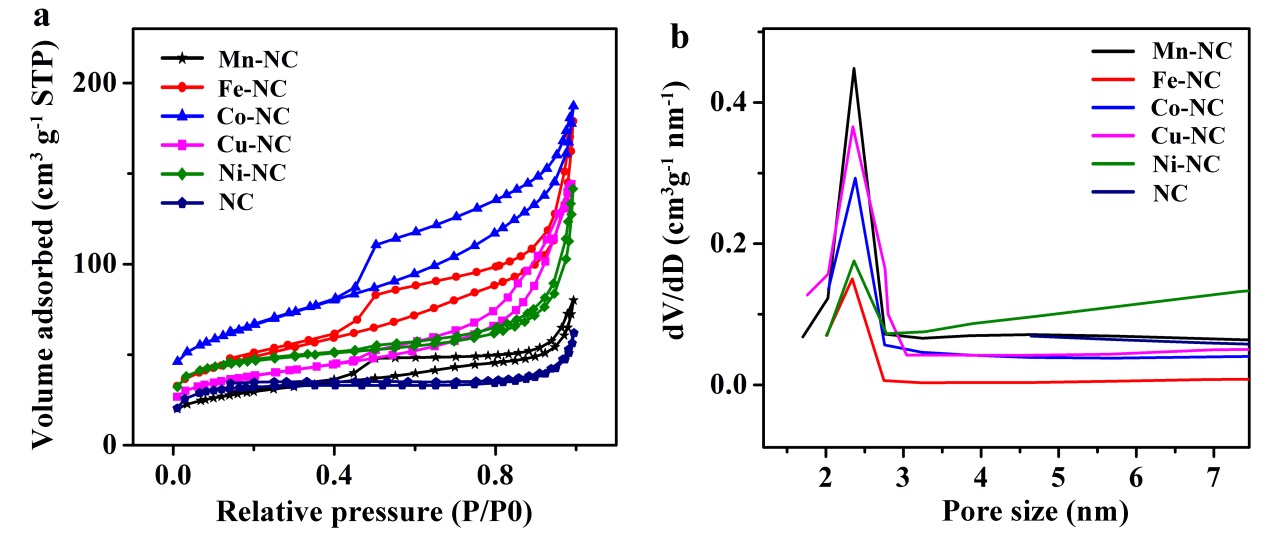


**Fig. S4** **a** N_2_ adsorption–desorption isotherms and **b** pore-size distribution of different samples.


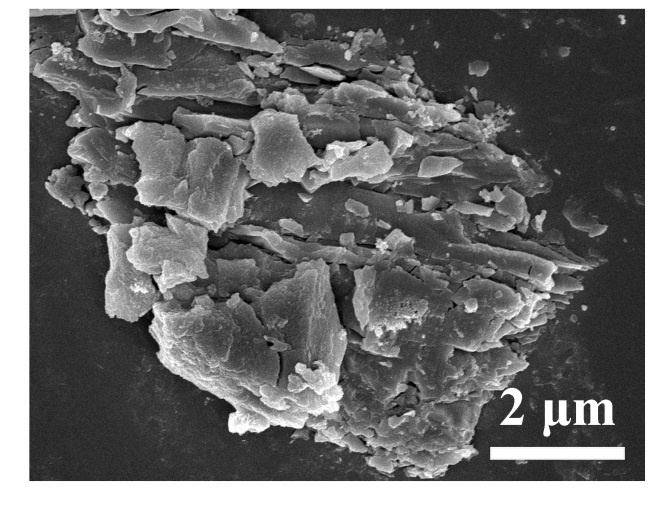


**Fig. S5** SEM images of the Mn–N_x_C-w.


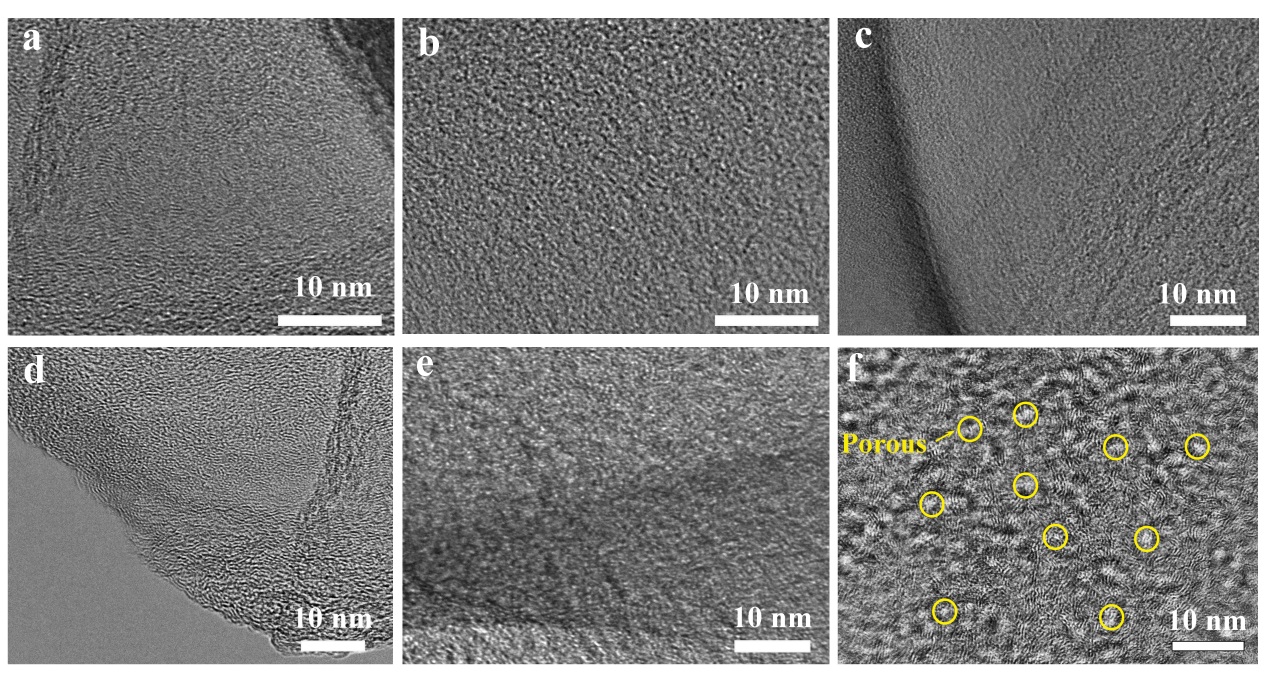


**Fig. S6** HRTEM images of **a** 3D NC, **b** 3D Fe–NC, **c** 3D Cu–NC, **d** 3D Ni–NC and **e** 3D Co–NC, and **f** 3D Mn–NC.


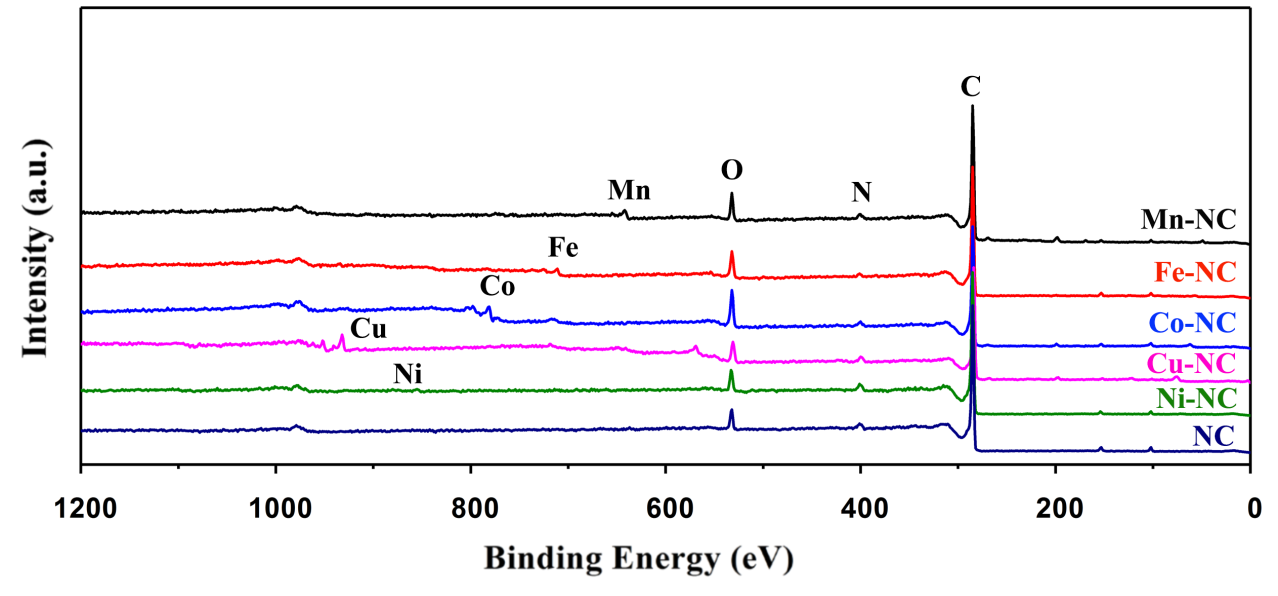


**Fig. S7** X-ray photoelectron spectroscopy (XPS) patterns of 3D NC, 3D Ni–NC, 3D Cu–NC, 3D Co–NC, 3D Fe–NC, and 3D Mn–NC.


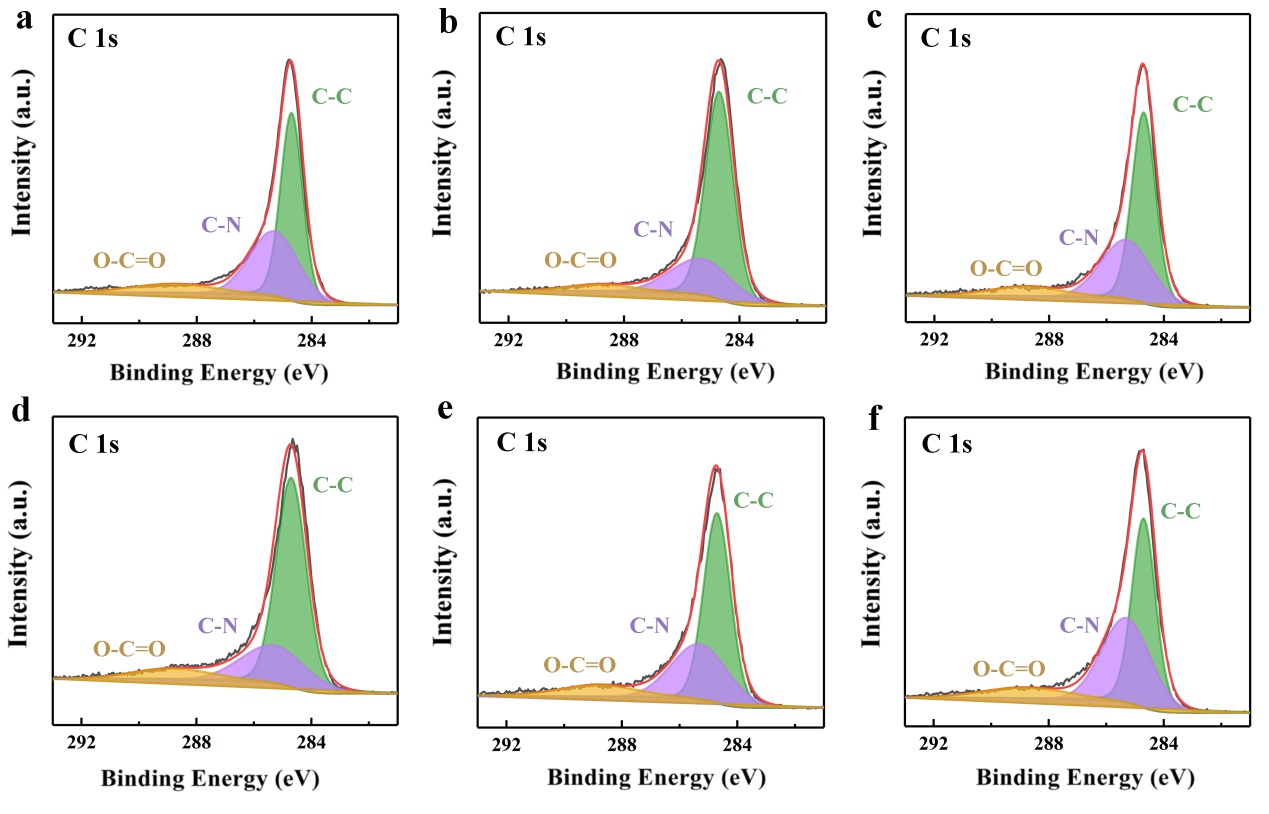


**Fig. S8** C 1s XPS spectra of **a** 3D NC, **b** 3D Mn–NC, **c** 3D Fe–NC, **d** 3D Co–NC, **e** 3D Cu–NC, and **f** 3D Ni–NC.


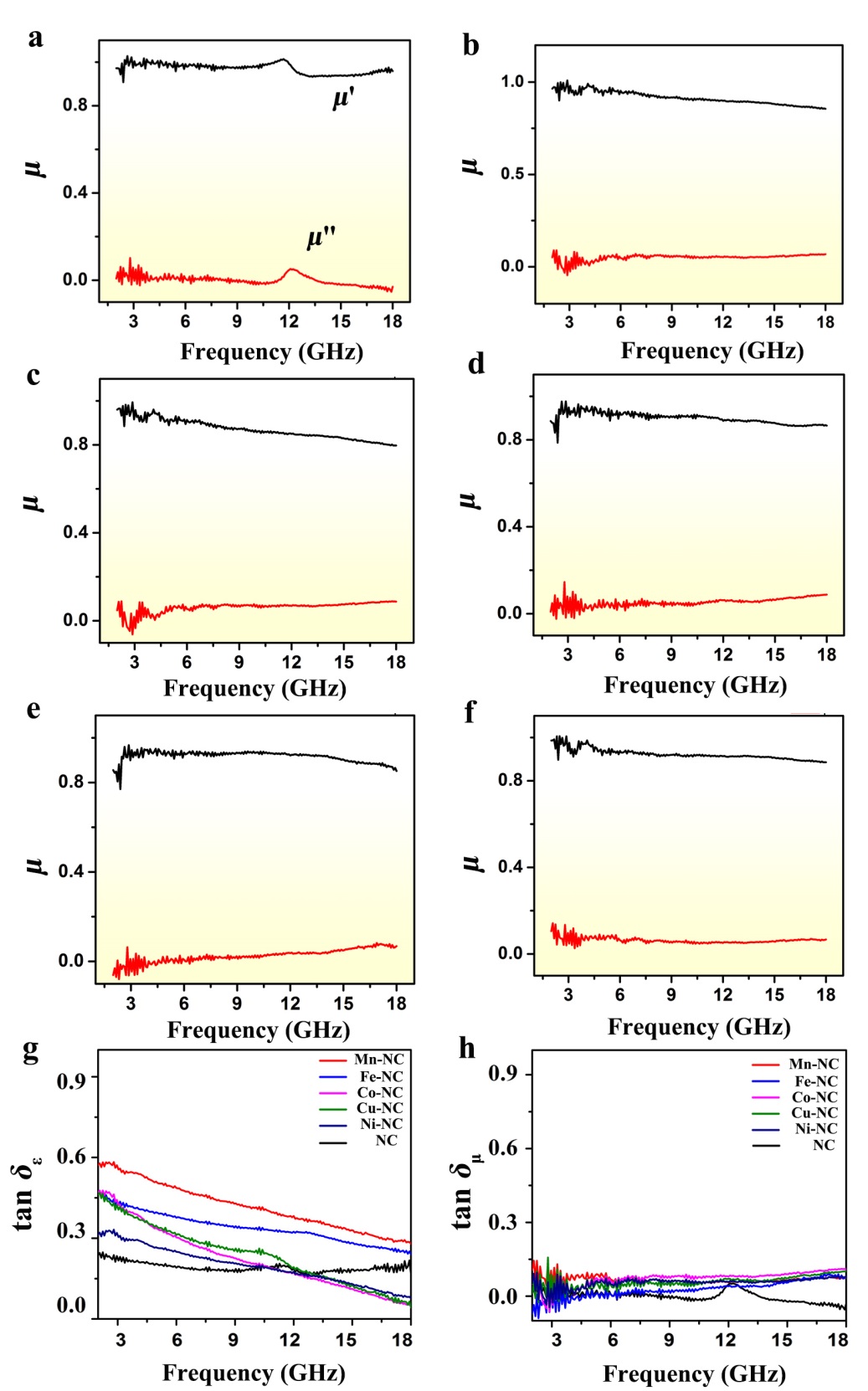


**Fig. S9** The relative real parts, imaginary parts of the complex permeability of **a** 3D NC, **b** 3D Ni–NC, **c** 3D Cu–NC, **d** 3D Co–NC, **e** 3D Fe–NC, and **f** 3D Mn–NC. **g-h** Dielectric loss tangent and magnetic loss tangent of the samples.


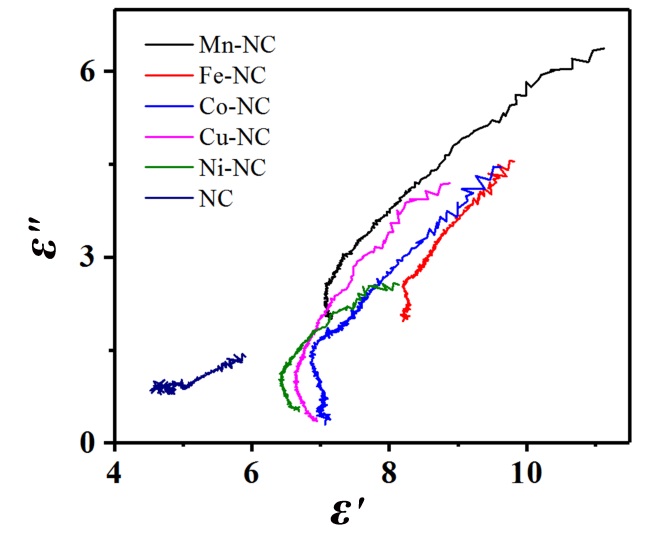


**Fig. S10** Cole–Cole plots of all the samples.


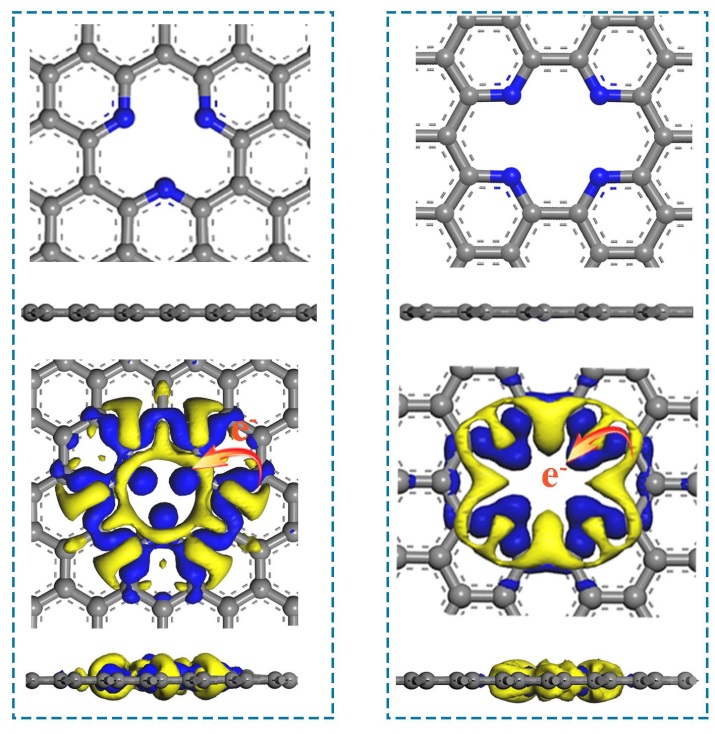


**Fig. S11** Calculated charge density difference of the different type N-doped sites in a single graphitic plane.


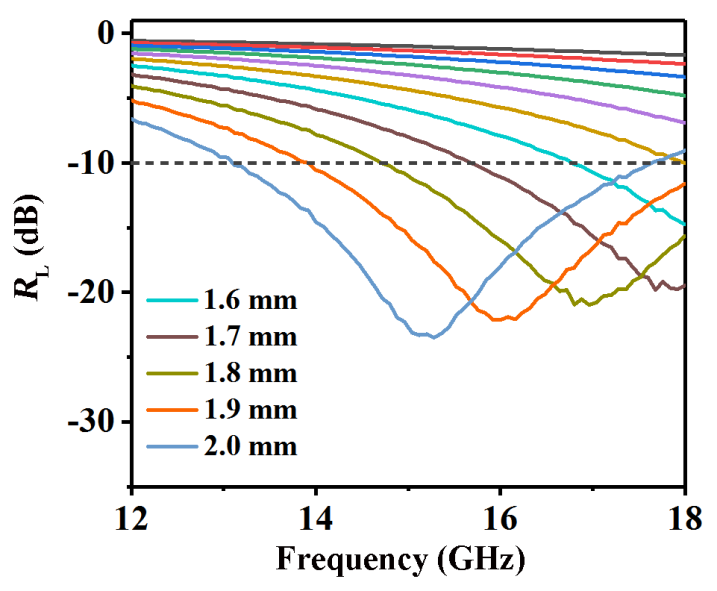


**Fig. S12** Reflection loss curves of 3D Mn–NC at the thickness < 2.0 mm.


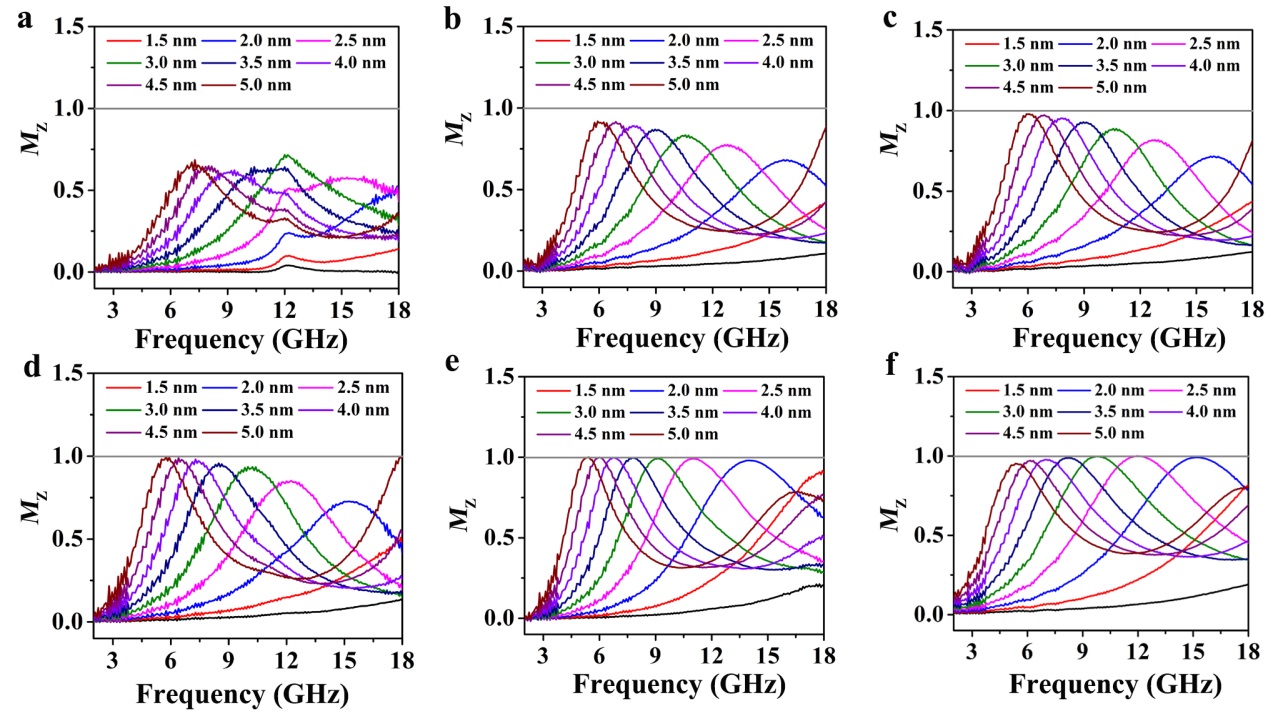


**Fig. S13** The degree of impedance matching of **a** 3D NC, **b** 3D Ni–NC, **c** 3D Cu–NC, **d** 3D Co–NC, **e** 3D Fe–NC, and **f** 3D Mn–NC.


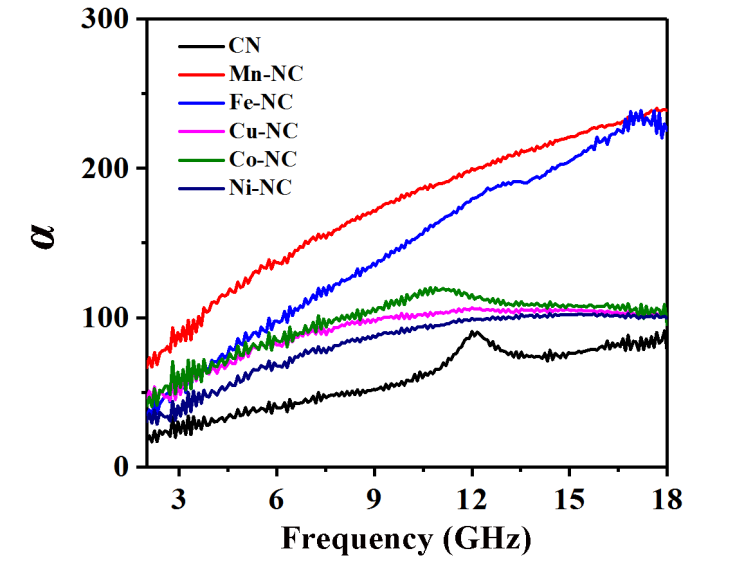


**Fig. S14** The *α*–*f* curves of all the samples.


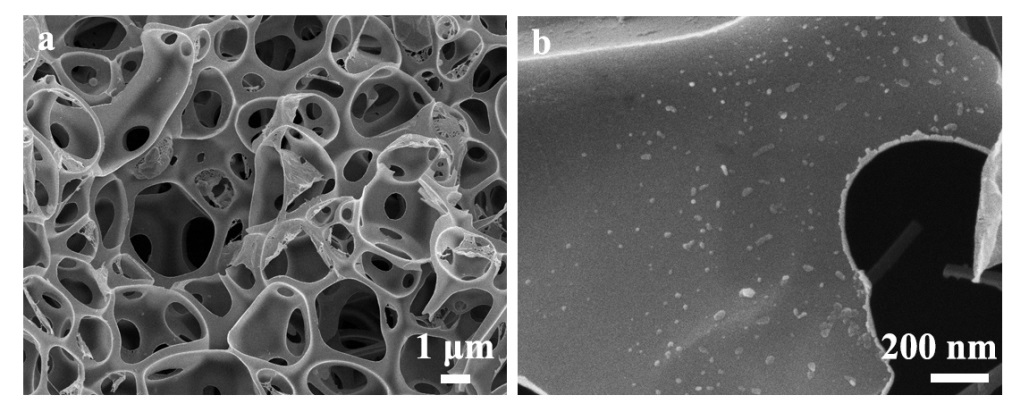


**Fig. S15** SEM images of the 3D Mn NPs–NC.


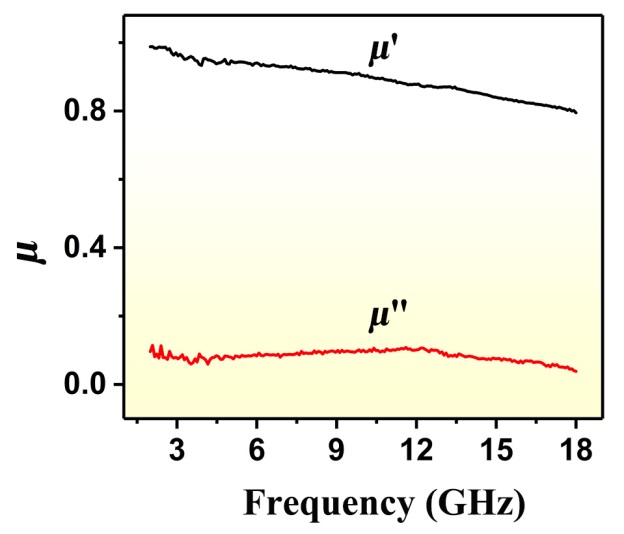


**Fig. S16** Frequency dependence of *µ*_r_ of 3D Mn NPs–NC.


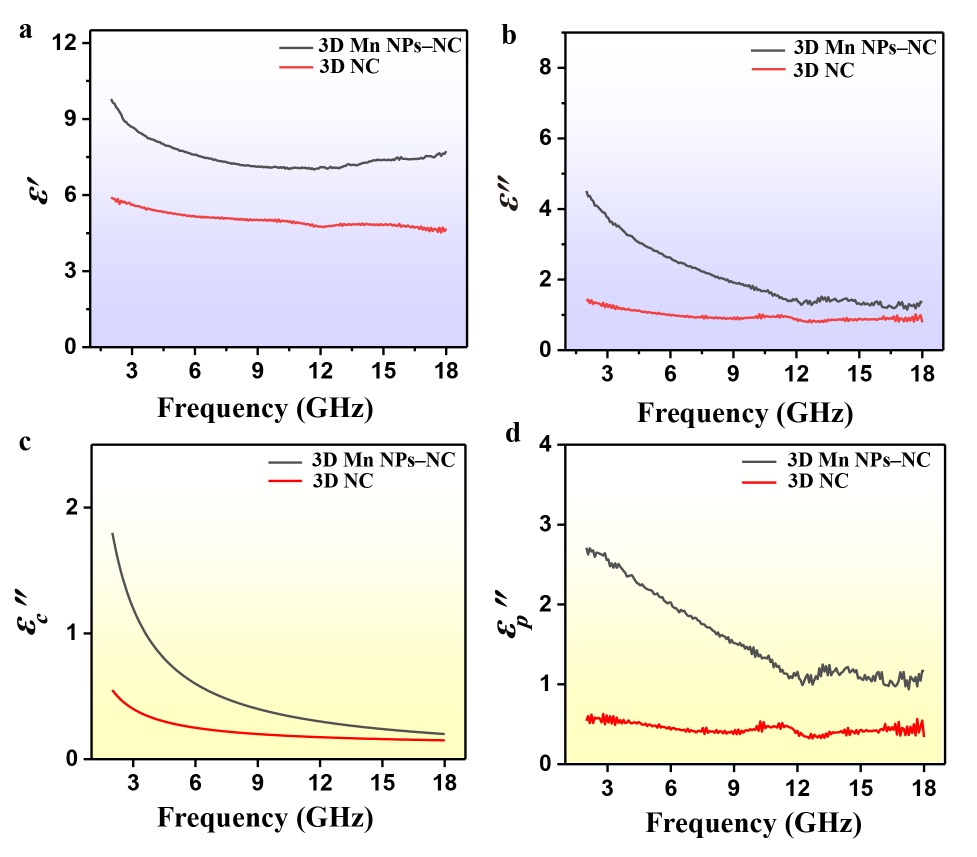


**Fig. S17 a–d** Frequency dependence of *ε*׳, *ε*״*, ε*_c_״ and *ε*_p_״ of 3D Mn NPs–NC and 3D NC.


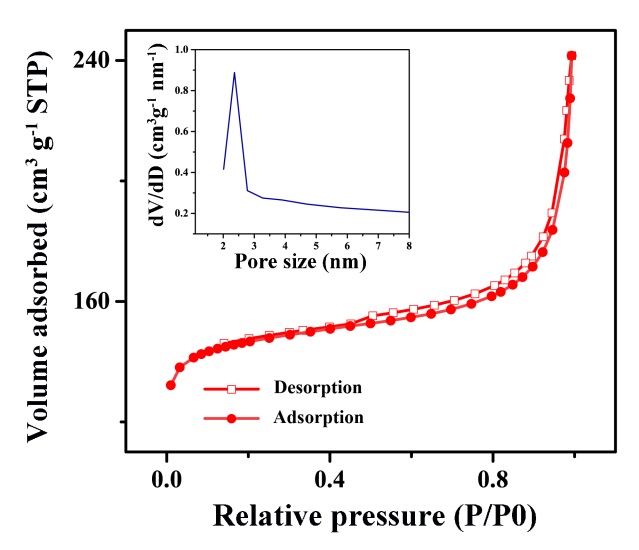


**Fig. S18** N_2_ adsorption–desorption isotherms of the 3D Mn NPs–NC.

**
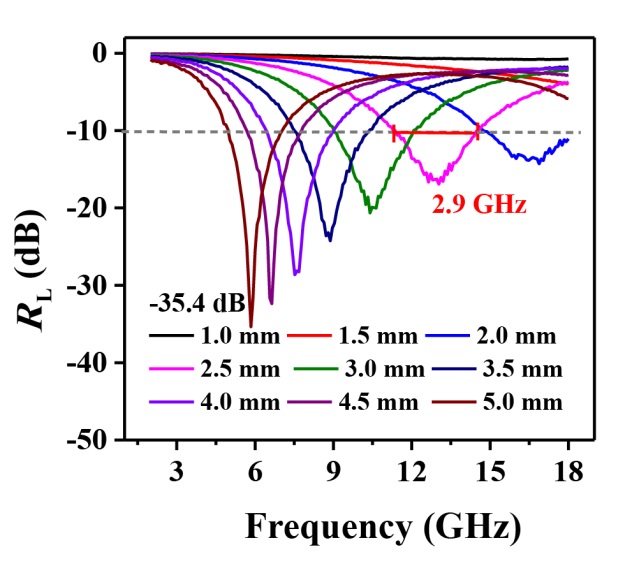
**

**Fig. S19** Reflection loss curves of the 3D Mn NPs–NC.


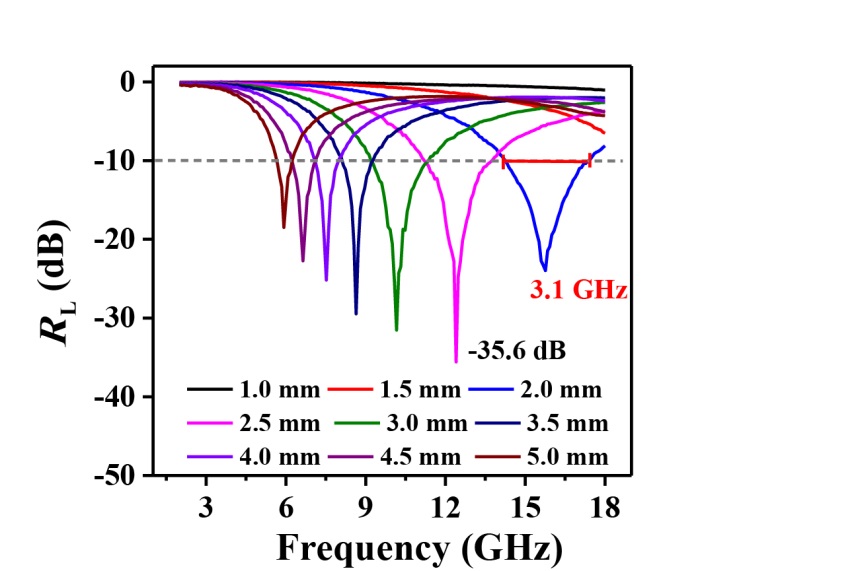


**Fig. S20** Reflection loss curves of the Mn–N_x_C-w.


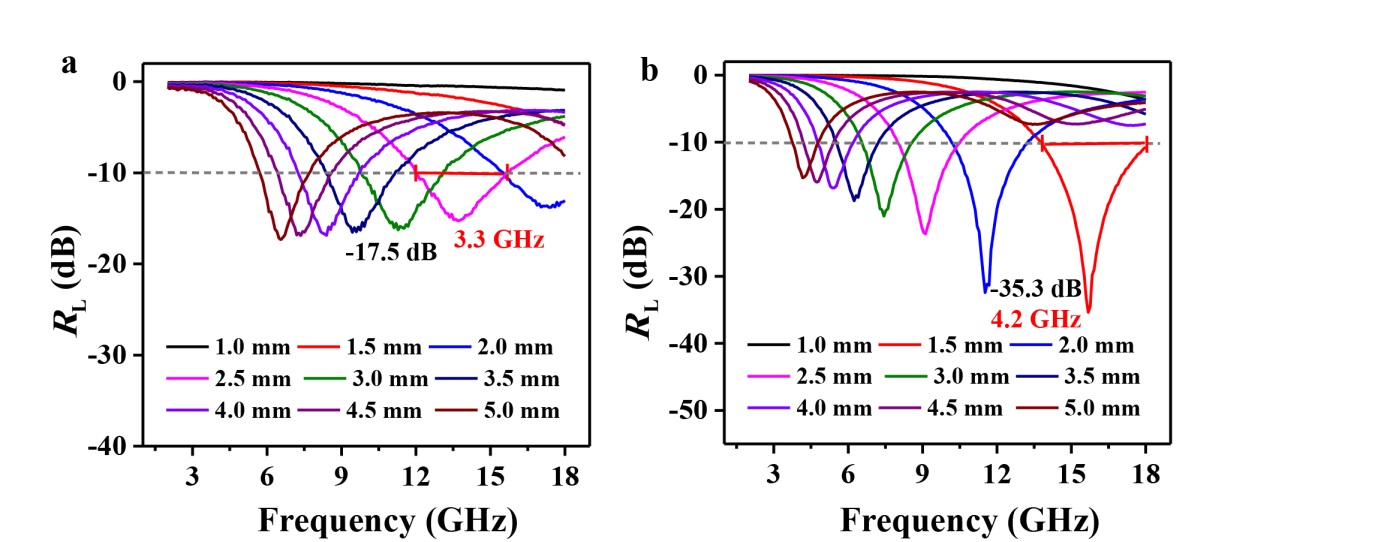


**Fig. S21** Reflection loss curves of the 3D Mn–NC with a filler loading of **a** 5 wt.%, **b** 15 wt.%.

**
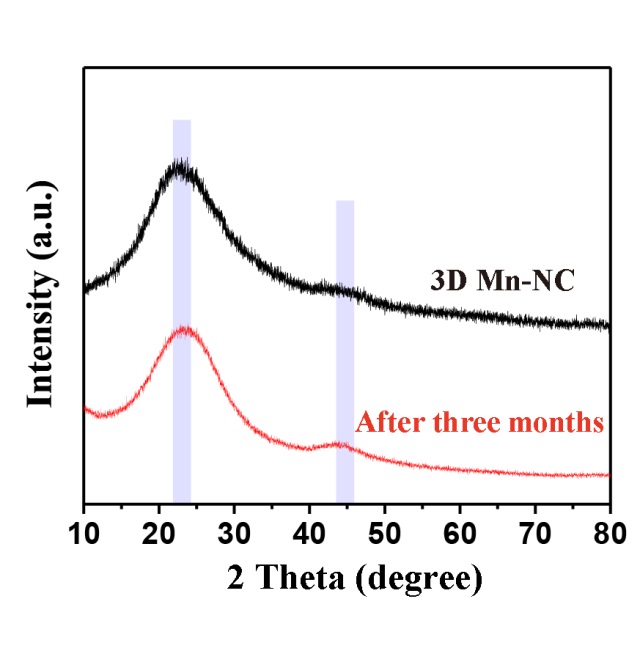
**

**Fig. S22** XRD patterns of the 3D Mn–NC and the 3D Mn–NC sample stored for three months.


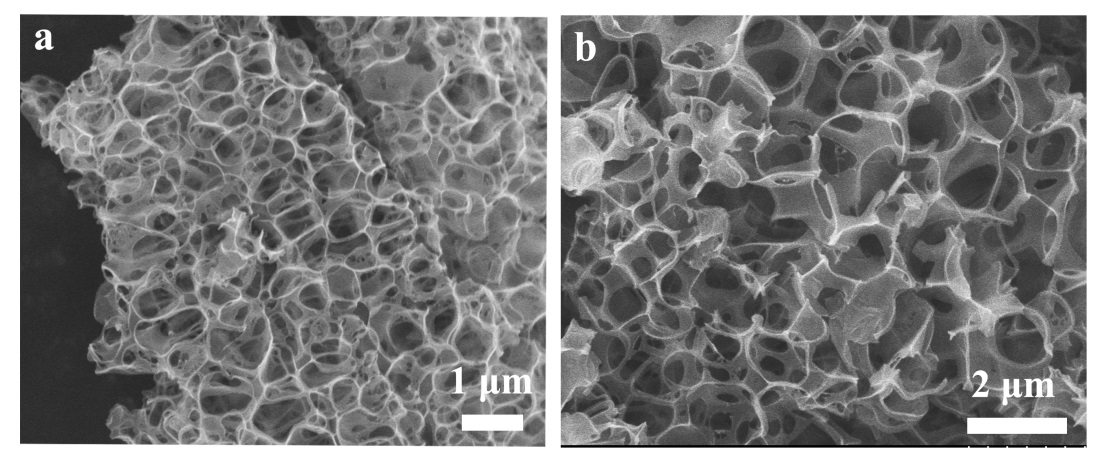


**Fig. S23** SEM images of **a** 3D Mn–NC and **b** 3D Mn–NC sample stored for three months.


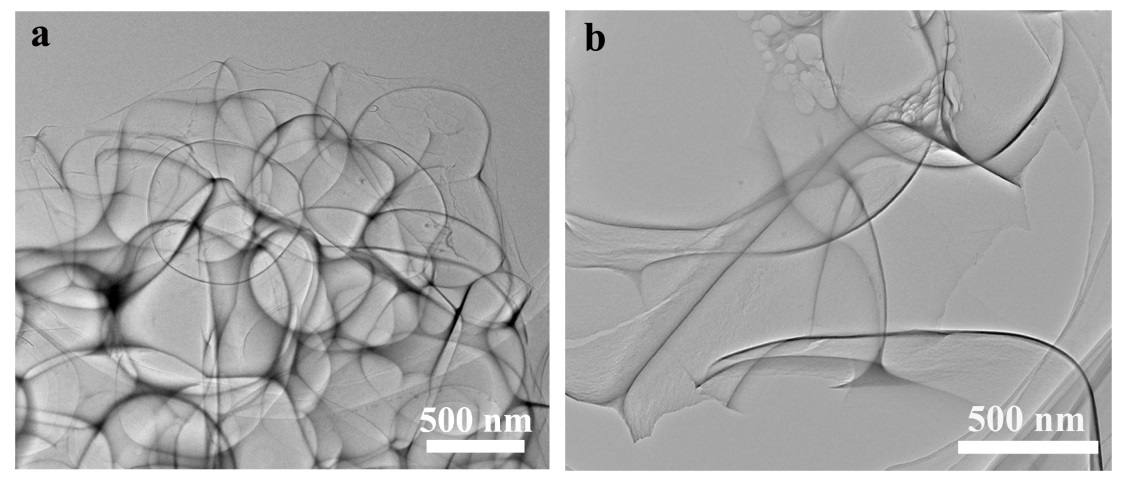


**Fig. S24** TEM images of **a** 3D Mn–NC and **b** 3D Mn–NC sample stored for three months.

**
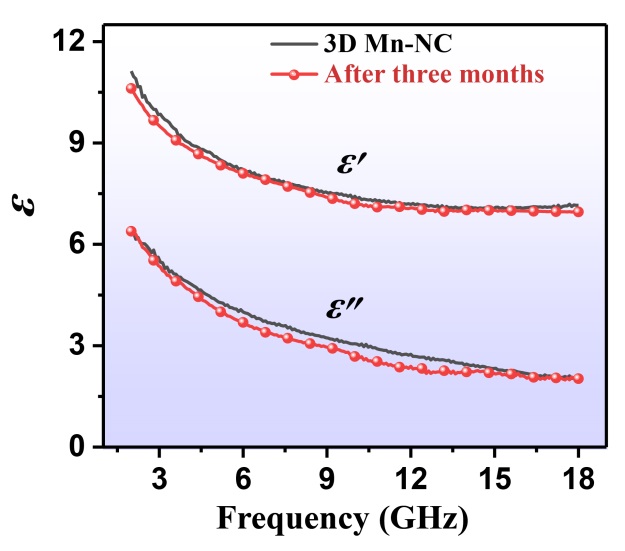
**

**Fig. S25** Frequency dependence of *ε*_r_ of the 3D Mn–NC and the 3D Mn–NC sample stored for three months.

**Table S1** BET specific surface area and pore size for all the samples.

|  | CN | Mn–NC | Fe–NC | Co–NC | Cu–NC | Ni–NC |
| --- | --- | --- | --- | --- | --- | --- |
| BET surface areas (m^2^/g) | 550 | 634 | 607 | 599 | 580 | 570 |
| Pore size (nm) | 2.35 | 2.32 | 2.37 | 2.35 | 2.33 | 2.35 |

**Table S2** Content of nitrogen species in the different samples.

|  | CN | Mn–NC | Fe–NC | Co–NC | Cu–NC | Ni–NC |
| --- | --- | --- | --- | --- | --- | --- |
| N (at.%) | 2.1 | 3.6 | 3.2 | 3.0 | 2.6 | 2.4 |

**Table S3** ICP-AES results of all the samples.

|  | Mn–NC | Fe–NC | Co–NC | Cu–NC | Ni–NC |
| --- | --- | --- | --- | --- | --- |
| Metal (wt.%) | 1.80 | 1.40 | 1.38 | 1.17 | 1.12 |

**Table S4** EXAFS fitting parameters at the Mn K-edge various samples (*Ѕ*_0_^2^=0.11).

| Sample | Path | C.N. | R (Å) | σ^2^×10^3^ (Å^2^) | ΔE (eV) | R factor |
| --- | --- | --- | --- | --- | --- | --- |
| Mn foil | Mn-Mn | 12* | 2.66±0.01 | 7.2±1.6 | 4.0±2.4 | 0.011 |
| MnO | Mn-O | 5.5±1.0 | 2.19±0.01 | 5.3±1.8 | 3.0±1.8 | 0.007 |
|  | Mn-Mn | 16.0±2.3 | 3.14±0.01 | 10.0±1.2 | 1.9±1.2 |  |
| Mn_2_O_3_ | Mn-O | 5.1±0.9 | 1.91±0.01 | 5.8±1.8 | -4.0±2.3 | 0.09 |
|  | Mn-Mn | 12.7±2.3 | 3.15±0.01 | 11.3±1.4 | 5.7±1.2 |  |
| Mn–NC | Mn-N | 3.8±1.9 | 2.16±0.04 | 7.6±8.8 | 8.9±2.7 | 0.016 |

*^a^C.N.*: coordination numbers; *^b^R*: bond distance; *^c^σ*^2^: Debye-Waller factors; *^d^* Δ*E*: the inner potential correction. *R* factor: goodness of fit. * The experimental EXAFS fit of metal foil by fixing CN as the known crystallographic value.

**Table S5** Electrical conductivity for all the samples.

|  | Mn–NC | Fe–NC | Co–NC | Cu–NC | Ni–NC | NC |
| --- | --- | --- | --- | --- | --- | --- |
| *σ* (S m^-1^) | 4.40 | 4.16 | 4.02 | 3.96 | 3.87 | 1.01 |

**Table S6** Mulliken charge (local of N_4_C and M-N_4_C structure) for all the samples.

|  | CN | Mn–NC | Fe–NC | Co–NC | Cu–NC | Ni–NC |
| --- | --- | --- | --- | --- | --- | --- |
| C | 0.18 | 0.11 | 0.11 | 0.11 | 0.11 | 0.11 |
| N | -0.33 | -0.43 | -0.47 | -0.46 | -0.49 | -0.45 |
| Metal |  | 1.17 | 1.23 | 1.18 | 1.42 | 1.20 |

**Table S7** Comparison of the EMW absorption performance of the previously reported carbon-based absorbers.

| Absorbers | *R*_L, min_  (dB) | Optimum  thickness  (mm) | Filler  loading  (wt. %) | *SRL*_l_  (dB·mg^-1^) | *SRL*_lt_  (dB·mm^-1^·mg^-1^) | Ref. |
| --- | --- | --- | --- | --- | --- | --- |
| Mn–NC | -46.2 | 2 | 10 | -4.62 | -2.31 | This work |
| GO–CNT–Fe_3_O_4_ | -37.25 | 5 | 30 | -1.24 | -0.25 | [s1] |
| FeCo–CNT | -46.5 | 1.7 | 50 | -0.93 | -0.55 | [s2] |
| C–Fe_2_O_3_–Fe_3_C–Fe–CNT | -42.6 | 3.5 | 20 | -2.13 | -0.61 | [s3] |
| Co–CNT–G | -65.6 | 2.19 | 30 | -2.18 | -0.99 | [s4] |
| Fe_3_O_4_–CNT–Carbon fibers | -50.9 | 2.5 | 25 | -2.03 | -0.81 | [s5] |
| Fe_3_O_4_–CNT | -43 | 1.5 | 30 | -1.43 | -0.95 | [s6] |
| Ferrite/Co/porous carbon | -47.3 | 2.5 | 70 | -0.68 | -0.27 | [s7] |
| Co–C | -62.12 | 2.4 | 30 | -2.07 | -0.86 | [s8] |
| Co@C microspheres | -68.7 | 1.65 | 70 | -0.98 | -0.59 | [s9] |
| CNT@TiO_2_ sponges | -31.8 | 2.0 | 30 | -1.06 | -0.53 | [s10] |
| FeCo/graphene hybrids | -40.2 | 2.5 | 50 | -0.80 | -0.32 | [s11] |
| CoFe@C | -43.5 | 2.5 | 50 | -0.87 | -0.34 | [s12] |

**References**

[s1] L. Wang, X. Jia, Y. Li, F. Yang, L. Zhang et al., Synthesis and microwave absorption property of flexible magnetic film based on graphene oxide/carbon nanotubes and Fe_3_O_4_ nanoparticles. J. Mater. Chem. A **2**, 14940−14946 (2014). <https://doi.org/10.1039/C4TA02815E>

[s2] B. Yang, Y. Wu, X. Li, R. Yu, Surface-oxidized FeCo/carbon nanotubes nanorods for lightweight and efficient microwave absorbers. Materials and Design **136**, 13−22 (2017). https://doi.org/10.1016/j.matdes.2017.09.055

[s3] B. Zhong, C. Wang, G. Wen, Y. Yu, L. Xia, Facile fabrication of boron and nitrogen co-doped carbon@Fe_2_O_3_/Fe_3_C/Fe nanoparticle decorated carbon nanotubes three-dimensional structure with excellent microwave absorption properties. Compos. Part B-Eng. **132**, 141−150 (2018). http://dx.doi.org/10.1016/j.compositesb.2017.09.001

[s4] X. Qi, Q. Hu, H. Cai, R. Xie, Z. Bai et al., Heteronanostructured Co@carbon nanotubes-graphene ternary hybrids: synthesis, electromagnetic and excellent microwave absorption properties. Sci. Rep. **6**, 37972 (2016). https://doi.org/10.1038/srep37972

[s5] J. Qiu, T. Qiu, Fabrication and microwave absorption properties of magnetite nanoparticle–carbon nanotube–hollow carbon fiber composites. Carbon **81**, 20–28 (2015). http://dx.doi.org/10.1016/j.carbon.2014.09.011

[s6] N. Li, G. Huang, Y. Li, H. Xiao, Q. Feng et al., Enhanced microwave absorption performance of coated carbon nanotubes by optimizing the Fe_3_O_4_ nanocoating structure. ACS Appl. Mater. Interfacs **9**, 2973−2983 (2017). https://doi.org/10.1021/acsami.6b13142

[s7] L. Wang, Y. Guan, X. Qiu, H. Zhu, S. Pan et al., Efficient ferrite/Co/porous carbon microwave absorbing material based on ferrite@metal–organic framework. Chem. Eng. J. **326**, 945–955 (2017). http://dx.doi.org/10.1016/j.cej.2017.06.006

[s8] K. Wang, Y. Chen, R. Tian, H. Li, Y. Zhou et al., Porous Co−C core−shell nanocomposites derived from Co-MOF-74 with enhanced electromagnetic wave absorption performance. ACS Appl. Mater. Interfaces **10**, 11333−11342 (2018). https://doi.org/10.1021/acsami.8b00965

[s9] D. Ding, Y. Wang, X. Li, R. Qiang, P. Xu et al., Rational design of core-shell Co@C microspheres for high-performance microwave absorption. Carbon **111**, 722−732 (2017). http://dx.doi.org/10.1016/j.carbon.2016.10.059

[s10] C. Mo, R. Yang, D. Lu, L. Yang, Q. Hu et al., Lightweight, three-dimensional carbon nanotube@TiO_2_ sponge with enhanced microwave absorption performance. Carbon **144**, 433−439 (2019). https://doi.org/10.1016/j.carbon.2018.12.064

[s11] X. Li, J. Feng, Y. Du, J. Bai, H. Fan et al., One-pot synthesis of CoFe_2_O_4_/graphene oxide hybrids and their conversion into FeCo/graphene hybrids for lightweight and highly efficient microwave absorber. J. Mater. Chem. A **3**, 5535–5546 (2015). https://doi.org/10.1039/C4TA05718J

[s12] X. Zeng, B. Yang, L. Zhu, H. Yang, R. Yu, Structure evolution of prussian blue analogues to CoFe@C core–shell nanocomposites with good microwave absorbing performances. RSC Adv. **6**, 105644–105652 (2016). <https://doi.org/10.1039/C6RA18928H>
